# Metal‐Mediated Nitrogen Doping of Carbon Supports Boosts Hydrogen Production from Ammonia

**DOI:** 10.1002/anie.202522937

**Published:** 2025-12-21

**Authors:** Thomas J. Liddy, Benjamin J. Young, Emerson C. Kohlrausch, Andreas Weilhard, Gazi N. Aliev, Yifan Chen, Manfred E. Schuster, Mohsen Danaie, Luke L. Keenan, Donato Decarolis, Diego Gianolio, Siqi Wang, Mingming Zhu, Graham J. Hutchings, David M. Grant, Wolfgang Theis, Tien‐Lin Lee, David A. Duncan, Alberto Roldan, Andrei N. Khlobystov, Jesum Alves Fernandes

**Affiliations:** ^1^ School of Chemistry University of Nottingham Nottingham NG7 2RD UK; ^2^ School of Physics and Astronomy University of Birmingham Edgbaston B15 2TT UK; ^3^ Johnson Matthey Technology Centre Blounts Court Sonning Common RG4 9NH UK; ^4^ Electron Physical Science Imaging Centre (ePSIC) Harwell Science & Innovation Campus Didcot OX11 0DE UK; ^5^ Diamond Light Source Ltd. Harwell Science & Innovation Campus Didcot OX11 0DE UK; ^6^ Faculty of Engineering and Applied Sciences Cranfield University College Road, Wharley End Bedford MK43 0AL UK; ^7^ Cardiff Catalysis Institute School of Chemistry Translational Research Hub Cardiff University Cardiff CF24 4HQ UK; ^8^ Advanced Materials Research Group Faculty of Engineering University of Nottingham Nottingham UK

**Keywords:** Ammonia decomposition, Heterogeneous catalysis, Hydrogen, N‐doped carbon, Ruthenium

## Abstract

Ammonia is an attractive hydrogen carrier, yet its practical use is limited by the need for efficient catalytic decomposition. We demonstrate that in‐situ N‐doping of Ru nanoparticles and graphitized carbon nanofiber supports during reaction produces a sharp increase in hydrogen production during the first 40 h, followed by stable activity. Spectroscopic and microscopic analyses, together with density functional theory simulations, reveal that Ru nitridation is rapid and support‐independent, resulting in a mechanistic shift from the traditional Langmuir–Hinshelwood to a Mars–van Krevelen pathway, further confirmed by isotopic labelling experiments. In contrast, the progressive nitridation of the carbon support, observed via X‐ray photoelectron spectroscopy, modulates the electronic environment of Ru and functions as a dynamic nitrogen reservoir that enables reversible N atoms exchange with the Ru particles, facilitating N desorption from the Ru surface and thereby governing the catalytic activity enhancement. These new findings provide new mechanistic insight into ammonia decomposition and establish progressive nitrogen doping of carbon supports as a strategy for designing efficient metal‐based catalysts for hydrogen production.

## Graphical abstract

A virtuous catalytic cycle is established as the carbon support simultaneously tunes electron density and stores atomic nitrogen formed via Ru–N species during ammonia decomposition, resulting in self‐enhancing catalysis.

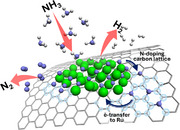

Hydrogen is a key green energy vector due to its high energy content and clean combustion. However, its low volumetric energy density and difficult storage limit large‐scale use. Ammonia, which contains more hydrogen per unit volume than molecular hydrogen at standard temperature and pressure, is a promising alternative that can be stored at moderate pressure and room temperature with well‐established storage and transport infrastructure. These properties make it attractive for hydrogen storage, though efficient catalytic cracking remains a major barrier.^[^ ^1^, ^2^, ^3^ ^]^

Industrial cracking typically employs Ni or Fe catalysts at 600–700 °C, while Ru catalysts achieve similar activity at about 450 °C.^[^ ^4^ ^]^ Despite its superior performance, the high cost of Ru necessitates careful optimization. The exceptional activity of Ru can be rationalized by the Sabatier principle, which relates the thermodynamics of intermediate binding to kinetic barriers through the Bell–Evans–Polanyi relationship.^[^ ^5^, ^6^, ^7^, ^8^, ^9^ ^]^ Ammonia decomposition can be simplified into three key steps: adsorption, dehydrogenation, and recombinative desorption of H_2_ and N_2_.^[^ ^10^, ^11^, ^12^ ^]^ The rate‐determining step (RDS) depends on the binding strength of surface nitride (N*). Noble metals are typically limited by the NH_3_ adsorption and N─H bond scission at high temperatures (>500 °C), whereas non‐noble metals are constrained by N* recombination at lower temperatures.^[^ ^13^, ^14^, ^15^, ^16^, ^17^, ^18^ ^]^ For Ru, the RDS has remained somewhat ambiguous but is generally considered to involve recombinative N_2_ desorption,^[^ ^19^, ^20^, ^21^ ^]^ consistent with its position near the apex of the volcano plot and its correspondingly high intrinsic activity.^[^ ^14^ ^]^ Previous studies have shown that tailoring the local environment of Ru, for example, by using nitrogen‐doped carbon supports, significantly improves hydrogen production.^[^ ^22^, ^23^, ^24^, ^25^, ^26^, ^27^ ^]^ However, the mechanistic role of N doping remains unclear, particularly regarding how it affects the binding of Ru–NH*_x_* species and thus the overall catalytic performance. Moreover, most studies attribute higher activity mainly to stabilization of Ru particles at N sites, without fully addressing the interplay between particle stabilization and electronic modification.

Herein, we demonstrate in‐situ N‐doping of Ru nanoparticles and graphitized carbon nanofiber (GNF) support during ammonia decomposition, resulting in a sharp increase in hydrogen production over the initial 40 h, followed by stabilization. We decoupled the effects of Ru nitridation and carbon nitridation, showing that Ru nitridation occurs independently of the support, while progressive nitridation of the carbon support influences the Ru electronic environment, promotes N exchange between GNF and Ru, modulates Ru–N binding strength, and ultimately controls the catalytic activity enhancement. Isotopic labelling was further employed to establish that the catalytic cycle follows a Mars‐van Krevelen pathway. This study provides a framework for understanding how support nitridation influences Ru–N binding and for developing more efficient catalysts for hydrogen production from ammonia.

A flux of Ru atoms generated by magnetron sputtering was deposited onto graphitized GNFs,^[^ ^28^, ^29^ ^]^ where they migrated to defects such as step edges or other Ru atoms assembling into 2D‐like clusters with footprints of 6–7 nm^2^ (Figure 1a). Ru/GNF catalytic testing was conducted following an H_2_ reduction step at 450 °C for 1 h, followed directly by ammonia decomposition at 450 °C using 5% NH_3_ in argon. The Ru/GNF exhibited a sharp and unusual increase in H_2_ production over the first 30–40 h, stabilizing thereafter for at least 100 h (Figure 1b). This increase in activity was accompanied by a drop in the apparent activation energy (E_a_) across the entire reaction, from 65 to 55 ± 0.7 kJ mol^−1^, indicating an improved electronic environment for the catalyst (Figures 1b and S1). To investigate this behavior, we systematically investigated key catalyst properties, before and after reaction, including Ru crystallinity, particle size, local coordination, and electronic structure.^[^ ^30^, ^31^ ^]^

**Figure 1:**
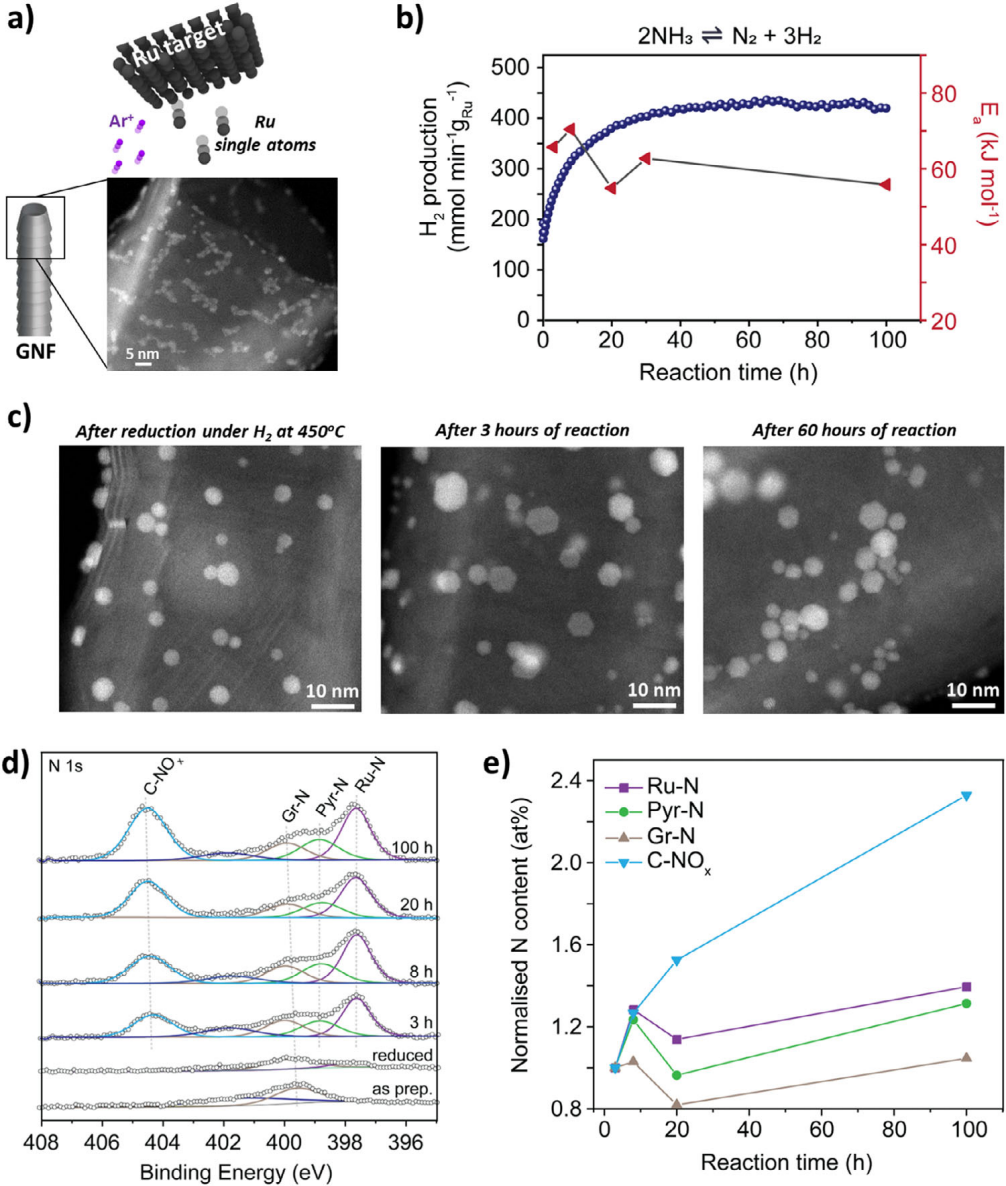
Preparation, catalytic performance, and characterization of Ru/GNF. a) Schematic of Ru atom deposition on graphitized nanofibers with corresponding electron microscopy image. b) Hydrogen production rate over 100 h of reaction (blue) and apparent activation energy (red) after 3, 8, 20, 30, and 100 h. c) AC‐STEM images of Ru/GNF after reduction, after 3 h, and after 60 h of reaction. d) and e) XPS of Ru/GNF as‐prepared, after reduction, and after 3, 8, 20, and 100 h of reaction.

AC‐STEM images revealed that, after reduction at 450 °C, the initial chain‐like Ru assemblies transformed into more 3D rounded particles with a mean diameter of 3.8 ± 0.9 nm (Figures 1a,c and S2). After 3 and 60 h under reaction conditions, a slight increase in mean particle size was observed to 4.7 ± 2.0 nm and 4.4 ± 1.6 nm, respectively, along with a transition to more faceted hexagonal closed‐packed (hcp) structure when compared to Ru/GNF after reduction (Figure 1c). Notably, no significant structural differences were observed between 3 h, when the reaction rate was still sharply increasing, and 60 h, when the rate had plateaued (Figures S2–S5). *In‐operando* gas‐cell AC‐STEM confirmed both the evolution from 2D to 3D morphologies and a slight increase in particle size of Ru/GNF upon switching from reduction to NH_3_ introduction (Figure S6). This indicates that particle restructuring is not the primary driver of the unusual activity increase, although it may contribute to the observed behavior.

X‐ray absorption near‐edge structure (XANES) measurements at the Ru K‐edge showed that as‐prepared Ru/GNF exhibited a higher white‐line (H_w_) intensity than Ru foil, indicating oxidation of Ru particles upon exposure to air (Figure S7a). In contrast, the reduced and post‐reaction catalysts (at 3, 8, 20, and 100 h) displayed significantly lower H_w_ intensities, close to Ru foil, consistent with a more metallic character. Extended X‐ray absorption fine structure (EXAFS) analysis confirmed this trend with the post‐reduction and post‐reaction catalysts resembling bulk Ru, whereas the as‐prepared sample showed an additional peak at ∼1.5 Å, ascribed to Ru─O and Ru─C bonds arising from the 2D‐like cluster structure, as well as a peak shifted by 0.3 Å relative to bulk Ru, which can be assigned to the second‐shell Ru─O─Ru coordination (Figure S7b). These results demonstrate that Ru was not oxidized upon air exposure, either after reduction or after catalysis. This can be ascribed to strong adsorption of hydride or nitride species on its surface, or to particle growth leading to a decrease in the number of Ru surface atoms.

To further assess the evolution of N species, X‐ray photoelectron spectroscopy (XPS) of the N 1s region was performed on bare GNF, Ru/ GNF as‐prepared, Ru/GNF after H_2_ reduction, and Ru/GNF after 3, 8, 20, and 100 h of reaction (Figure 1d). Bare GNFs showed no detectable N (Figure S8), whereas after deposition of Ru on GNF, either as prepared or after reduction, N species were observed. Under reaction conditions, significant Ru–N species (397.6 eV) appeared within 3 h, after which their intensity remained constant thereafter (Figure 1e), indicating rapid establishment of a steady‐state composition. While pyridinic N (398.8 eV) and graphitic N (400.0 eV) species remained largely unchanged, NO_x_ species (404.4 eV) increased steadily (Figure 1e). We attribute the NO*_x_* signal to pyridinic N formed during the reaction that becomes oxidized during transfer from the reactor to the XPS chamber. Although the formation of NO*_x_* is an *ex*‐*situ* artefact, its progressive increase still serves as a valuable probe for tracking nitridation of the support. Notably, the increase in catalytic activity parallels the sharp rise of NO*_x_* content over the 100 h reaction (Figure 1e), suggesting that N‐doping of the GNF support is central to the activity enhancement. Comparison with Ru/La_2_O_3_ reinforced this conclusion: although Ru nitridation (397.6 eV) was observed, NO*_x_* species (404.4 eV) were absent, and no sharp activity increase occurred, instead showing a slight deactivation (Figures S8b and S9). Additionally, the E_a_ for Ru/La_2_O_3_ exhibited a slight increase, from 72.0 ± 5.6 kJ mol^−1^ after 3 h to 78.1 ± 4.0 kJ mol^−1^ after 10 h, which may be related to the minor deactivation. Taken together, these results indicate that Ru nitridation is a rapid and support‐independent process, whereas GNF nitridation evolves more slowly and plays a central role in the observed activity enhancement.

To investigate the effect of N doping on Ru/GNF and its influence on the electronic properties of the catalytic system, H_2_‐temperature‐programmed surface reaction (TPSR) experiments were performed. H_2_‐TPSR profiles (Figure 2a) collected after 24 and 48 h revealed a shift of the maximum desorption temperature to lower values with extended reaction time, indicating more reactive N* species. Assuming a pseudo first‐order desorption of NH*_x_*‐containing species, the activation energy of desorption decreased by 14 kJ mol^−1^ (Table S1), highlighting a continuous evolution of the electronic environment around active sites during reaction.^[^ ^32^, ^33^ ^]^ These changes directly impact the reactivity of the crucial N* intermediate formed from NH_3_ on the Ru nanoparticle surface, leading to progressively weaker N* binding in accordance with the Bell–Evans–Polanyi principle.^[^ ^6^, ^34^, ^35^ ^]^

**Figure 2:**
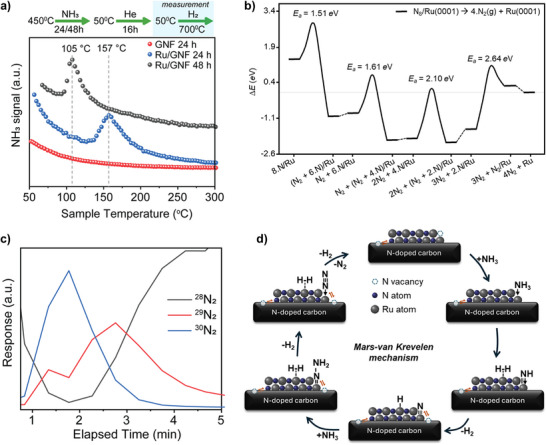
Mechanistic investigations of Ru/GNF. a) H_2_‐TPSR profiles of blank GNF and Ru/GNF. The catalysts were exposed to NH_3_ for 24 or 48 h, cooled to 50 °C under He flow for 16 mi, and then subjected to a flow of H_2_ while the temperature was increased from 50 to 700 °C, with NH_3_ desorption monitored. The full temperature range is shown in Figure S13. b) Energy profile for the nitrogen recombination and evolution from Ru(0001) slab. The dotted horizontal line across the profile indicates the reference energy (Δ*E* = 0 eV) of four isolated N_2_ molecules and a naked Ru surface. Transition states are represented by curved lines and their inset activation energies. Dashed lines between intermediates represent the desorption of N_2_ from the surface. The numerical data of all the reaction steps are summarized in Table S3. c) Isotopic scrambling experiments were performed by exposing Ru/GNF to ^14^NH_3_ at 450 °C for 60 h, followed by He flush for 16 min, and subsequent introduction of ^15^NH_3_ for 30 min. The measurement shown in this figure begins upon switching back to^14^NH_3_. Multiple cycles between ^14^NH_3_ and ^15^NH_3_ with He flushing in between, were then conducted (Figure S14). d) Proposed lattice‐N‐assisted Mars–van Krevelen mechanism for NH_3_ decomposition on Ru/GNF.

Results from the TPSR were complemented by periodic density functional theory (DFT) simulations of nitrogen recombination and desorption on the hexagonal close‐packed (hcp) Ru(0001) surface using the Vienna ab initio simulation package (VASP). Figures S10–S12 display the most energetically favorable configurations, which contain up to 9 nitrogen atoms, among the 48 configurations examined. The preferred adsorption site for atomic nitrogen is on the hollow FCC sites. We also evaluated potential subsurface nitrogen incorporation as a precursor to Ru nitridation by simulating 34 different arrangements where a single N atom was placed between the first and second top atomic layers while the rest of the N^*^ remain on the surface. Under the top Ru surface, atomic nitrogen sits preferentially on the HCP cavities.

Figure 2b indicates relative energies between N adsorption and lattice inclusion as a function of the number of surface nitrogens in the most energetically favorable configurations. Lattice nitrogen atoms have not been considered because they are less favorable than surface‐adsorbed ones. The first observation is that atomic N adsorbs exothermically, but it becomes less favorable as the nitrogen coverage increases. Conversely, stabilizing an N atom in the Ru lattice is unlikely at both low and high coverage. It is slightly favorable only at mid coverage, yet still less likely than remaining on the surface. It should be noted that the energies reported do not include entropic contributions. Overall, the energy profile shows that, starting from a high coverage (8/9 monolayers), an energy input of 1.51 eV will drive the exothermic recombination of atomic nitrogens to form N_2_ adsorbed on the surface. This can be achieved by increasing the temperature; the experiments are carried out at 450 °C. The desorption of the weakly adsorbed N_2_ is slightly unfavorable but can be easily surmounted at the working temperatures. The subsequent atomic nitrogen recombination is also thermodynamically favorable and has a similar activation energy. The formation of N_2_ at a coverage of 4/9 monolayers is practically isoenergetic, and therefore, thermodynamics will not drive the reaction. Furthermore, the recombination at medium coverage has an activation energy about 0.5 eV higher than at higher coverages. Recombining the last two nitrogen atoms on the Ru surface has a significant energy barrier of 2.6 eV. These thermodynamic and kinetic insights demonstrate how Ru nitridation enhances the nitrogen evolution process, a known rate‐limiting step in NH_3_ decomposition.

To further confirm the reaction mechanism, isotopic labelling experiments were carried out on Ru/GNF and Ru/1N‐GNF. The ^14^N‐doped GNF support was prepared in advance and used as a control in the scrambling experiments (Figure S15 and ESI method section).^[^ ^28^ ^]^ These experiments demonstrate that nitridated Ru/GNF follows a Mars–van Krevelen type mechanism (Figure 2c,d), resembling an inverted version previously reported for Co_3_Mo_3_N.^[^ ^36^ ^]^ Using Ru/GNF, when the feed is switched from ^15^NH_3_ to ^14^NH_3_, the evolution of ^30^N_2_ and ^29^N_2_ is observed, providing strong evidence that large amounts of ^15^NH*_x_* (*x* = 0–3) species are stored on the Ru particles. These species display remarkable stability, as flushing the sample with He at 450 °C for up to 16 min does not deplete ^15^N from the surface (Figure 2c). In contrast, switching from a ^15^NH_3_ feed to ^28^N_2_ does not lead to detectable ^29^N_2_ formation under either isothermal or isobaric TPSR conditions, indicating that no Ru surface sites were available for ^28^N_2_ cleavage (Figure S17). Using only ^15^NH_3_ over Ru/N‐GNF, the evolution of ^29^N_2_ is detected, which must be attributed to the transfer of ^14^N from the N‐GNF lattice to the Ru surface, where it subsequently reacts with ^15^NH_3_ to form ^29^N_2_ (Figures S16a). This demonstrates that N incorporated within the GNF structure participates in the mechanism. We therefore performed an additional control scrambling experiment with ^15^NH_3_ over ^14^N‐doped GNF in the absence of Ru nanoparticles, in which no scrambling was observed (Figures S16b). This control experiment confirms that the catalytic transformation must be assigned to the Ru nanoparticles, which establish a quasi‐equilibrium with N in the GNF lattice. In addition to isotopic labelling, the reaction order in NH_3_ was determined to be 0.7 (Figure S18), further supporting a Mars–van Krevelen–type mechanism. Taken together, these findings indicate that surface nitrides (N*) react with gaseous NH_3_ to form surface N_2_H*_x_* intermediates,^[^ ^36^ ^]^ which subsequently undergo dehydrogenation to release H_2_ and N_2_ (Figure 2d). These results reveal that the catalytic cycle proceeds via a Mars–van Krevelen type mechanism on the nitridated Ru/GNF, rather than through a conventional Langmuir–Hinshelwood pathway. Furthermore, the establishment of the Mars–van Krevelen mechanism appears to occur rapidly and is directly associated with the nitridation of the Ru particles, whereas the electronic modulation of Ru and the N exchange between Ru and N‐GNF evolve more slowly and correlate with the progressive nitridation of the GNF support. In this way, the nitride species on the Ru particles become destabilized by the nitridation of the support, leading to enhanced reactivity and sustained catalytic activity over extended time periods, as shown in Figure 1b.

In summary, we have demonstrated that progressive, in‐situ nitrogen doping of Ru nanoparticles and graphitized carbon nanofiber supports during ammonia decomposition leads to a significant increase in hydrogen production rate followed by long‐term stabilization. Spectroscopic and microscopic analyses and atomistic simulations show that Ru nitridation is rapid and support‐independent, resulting in a mechanistic shift from the traditional Langmuir–Hinshelwood pathway to a Mars–van Krevelen pathway, as confirmed by isotopic labelling experiments. In contrast to previously established mechanisms, the progressive nitridation of the carbon support modulates the Ru electronic environment and enables N exchange between Ru and N‐doped GNF, generating more reactive N species and thereby governing the catalytic activity enhancement. These findings advance mechanistic understanding of ammonia decomposition and establish progressive, in‐situ nitrogen doping of carbon supports mediated by metal particles as a strategy for designing efficient and durable Ru‐based catalysts for hydrogen production.

## Supporting Information

The Supporting Information is available. It includes AC‐STEM images, XPS, EXAFS, and catalytic experiments, along with complementary atomistic modelling data.

## Author Contributions

**T.L**.: Conceptualization; data curation; investigation; methodology; writing—original draft preparation; writing—review & editing; **B.Y**.: Conceptualization; data curation; investigation; methodology; **E.C.K**: data curation; investigation; methodology; **A.W**.: data curation; formal analysis; investigation; writing—review & editing; **G.A**.: data curation; formal analysis; **Y.C**: data curation; formal analysis; **M.E.S**.: data curation; formal analysis; **M.D**.: data curation; formal analysis; **L.L.K**.: data curation; investigation; **D.D**.: data curation; investigation; **D.G**.: data curation; investigation; methodology; **S.W**.: data curation; formal analysis; investigation; **M.Z**.: data curation; investigation; methodology; **G.H**.: original draft preparation; writing—review & editing; **W.T**.: data curation; formal analysis—review & editing; **T.L.L**.: data curation; investigation; methodology; **D.A.D**.: data curation; formal analysis; original draft preparation; writing—review & editing; **A.R**.: data curation; formal analysis; original draft preparation; writing—review & editing; **A.N.K**: conceptualization; funding acquisition; writing—review & editing; **J.A,F**: conceptualization; funding acquisition; methodology; supervision; writing—original draft preparation; writing—review & editing.

## Conflict of Interests

The authors declare no conflict of interest.

## Supporting information



Supporting Information

## Data Availability

The data that support the findings of this study are available from the corresponding author upon reasonable request.
