# Supplementary material for: Metal‐Mediated Nitrogen Doping of Carbon Supports Boosts Hydrogen Production from Ammonia
Source: Angew Chem Int Ed Engl. 2025 Dec 21;65(8):e22937. doi: 10.1002/anie.202522937 (PMC12910157; doi:10.1002/anie.202522937)
Supplement: Supplementary file 1 — Supporting Information [file ANIE-65-e22937-s001.docx]

Metal-Mediated Nitrogen Doping of Carbon Supports Boosts Hydrogen Production from Ammonia

Thomas J. Liddy^1,5^, Benjamin J. Young^1^, Emerson C. Kohlrausch^1^, Andreas Weilhard^1^, Gazi N. Aliev^2^, Yifan Chen^1^, Manfred E. Schuster^3^, Mohsen Danaie^4^, Luke Keenan^5^, Donato Decarolis^5^, Diego Gianolio^5^, Siqi Wang^6^, Mingming Zhu^6^, Graham J. Hutchings^7^, David M. Grant^8^, Wolfgang Theis^2^, Tien-Lin Lee^5^, David Duncan^1,5^, Alberto Roldan^7^, Andrei N. Khlobystov^1^ & Jesum Alves Fernandes^1^*

^1^School of Chemistry, University of Nottingham, NG7 2RD, UK.

^2^Nanoscale Physics Research Laboratory, School of Physics and Astronomy, University of Birmingham, Edgbaston, B15 2TT, UK.

^3^Johnson Matthey Technology Centre, Blounts Court, Sonning Common, RG4 9NH, UK.

^4^Electron Physical Science Imaging Centre (ePSIC), Harwell Science & Innovation Campus, Didcot, OX11 0DE, UK.

^5^Diamond Light Source Ltd., Harwell Science & Innovation Campus, Didcot, OX11 0DE, UK.

^6^Cranfield University, College Road, Wharley End, Bedford, MK43 0AL, UK.

^7^Cardiff Catalysis Institute, School of Chemistry, Translational Research Hub, Cardiff University, Cardiff, CF24 4HQ, UK.

^8^Advanced Materials Research Group, Faculty of Engineering, University of Nottingham, Nottingham, United Kingdom.

**Supporting Information**

**Catalysis:** Catalytic decomposition of NH_3_ was conducted in a packed bed reactor in which the reaction temperature was monitored by a thermocouple in the catalyst bed (Hiden CATLAB Microreactor). Typically, 2.5 mg of catalyst was loaded into a quartz tube (5 mm ID), packed with quartz wool to secure the catalyst in place. Prior to testing the catalyst was reduced in situ at 450°C (5 °C min^-1^ ramp) for 1 hr under a 25 mL min^-1^ flow of 5% H_2_ in Ar and 5 mL min^-1^ of He. The gas flow was switched to 25 mL min^-1^ of 5% NH_3_ in Ar and 5 mL min^-1^ of He. This gives a constant WHSV of the progress of the reaction was monitored by an in-line mass spectrometer (Hiden QGA), taking a reading every ~20 s. The output of the mass spectrometer was calibrated against known flows of gas (L min^-1^). The rate calculations were calculated using:

$Rate \left( mmol {min}^{-1}g_{Ru}^{-1} \right) = \frac{V_{H_{2}}P}{RTm}*1000 (Equation 1)$

Where $V_{H_{2}}$is the volumetric flow of hydrogen produced (L min^-1^), $P$ is ambient pressure (atm), $T$ is the reaction temperature (K), R is the ideal gas constant (0.08206 L atm mol^-1^ K^-1^) and m is the mass of ruthenium (g). The bare PR-24 GNF support was also confirmed to be inactive for ammonia decomposition. For activation energy calculations, the reactor was cooled in 10 °C steps from 450-400 °C. Activation energy calculations were made using the Arrhenius equation from the post-reaction downwards ramp data.

**Rate order of NH_3_:** 3 mg of Ru/GNF is activated under 25 ml min^-1^ 5% H_2_ in Ar and 5 mL min^-1^ He for 1h at 450°C. Afterwards the feed is changed to 25 ml min^-1^ 5% NH_3_ in Ar and 5 mL min^-1^ He for 40h. Then the NH3 feed is changed to 23 ml min^-1^, 21 ml min^-1^, 19 ml min^-1^ and 17 ml min^-1^ and the feed is made up with He to give a total feed of 30 mL min^-1^. The difference in the resulting rate of H_2_ production is then obtained by the difference in partial pressure of H_2_ in the exhaust.

**Scrambling with 15NH3 and N-doped Ru/GNF:** 30 mg Ru deposited onto N-doped GNF (Ru/GNF-N) or pure N-GNF is heated under a feed of 30 mL min^-1^ He to 200°C and after 1h cooled to 50°C. Afterwards the feed is switched to 25 mL min^-1^ ^15^NH_3_ and 5 mL min^-1^ He, and the sample heated to 600°C with a ramp rate of 8 K min^-1^. The evolution of ^30^N_2_, ^29^N_2_, and ^28^N_2_ is monitored using a quadrupole mass spectrometer.

**H_2_ – TPSR:** 30 mg of catalyst was loaded into a quartz tube (5 mm ID), packed with quartz wool to secure the catalyst in place. Prior to testing, the catalyst was treated as per the catalysis section for the desired space of time followed by a cool down to 50 °C at 5 °C min^-1^ under 25 mL min^-1^ NH_3_ and 5 mL min^-1^ He flow for 5 h. NH_3_ flow was then stopped and 30 mL min^-1^ He was flowed for 16 h. The temperature was then ramped at 8 °C min^-1^ to 700 °C under the same He flow and the desorbed NH_3_ was measured.

The desorption activation energy for H_2_-N*TPSR was calculated using the temperature $(T_{P})$ of the maximum desorption rate and applying the simplified Redhead equation. For the pre-exponential factor, A, a value of 10^13^ s^-1^ was assumed whilst the desorption order was assumed to be 1. The heating ramp, β, was 8 K min^-1^ (0.13 K s^-1^). The values for E_d_ can be found (Table S1) The simplified Redhead equation is:

$E_{d}=RT_{p}\left( \ln\left( \frac{AT_{p}}{\beta} \right)-3.64 \right) (Equation 2)$

**Table S1** Results of the H_2_-N*TPSR measurements. Desorption energies calculated using the simplified Redhead equation

| Entry | Reaction time | T_p_ (°C) | T_p_ (K) | E_d_ (kJ mol^-1^) |
| --- | --- | --- | --- | --- |
| 1 | 24 | 157 | 430.15 | 123 |
| 2 | 48 | 105 | 378.15 | 108 |

**Isotopic Scrambling:** 30 mg of catalyst was exposed to reaction conditions (see **Catalysis**) for 60 h under a flow of ^14^NH_3_ at 450 °C. Maintaining temperature, NH_3_ flow was stopped, and the system was flushed with He (10 mL/min) for 5, 7, 10, 13 and 16 minutes. After each flush, isotopically labelled ^15^NH_3_ was flowed (10 mL/min) for 30 minutes and the N_2_ species were measured. The reverse was also measured, from ^15^NH_3_ to He flush to ^14^NH_3_ and is shown in **Figure 2c and Figure S14**.

**Catalysts preparation:** GNFs were supplied by PyroGraf (PR-24-XT-HHT) with iron content below 100 ppm. All depositions were conducted using an AJA magnetron sputtering system. Briefly, the GNF (0.2 g) were placed in the glove box and heated under vacuum for 5 hours (100 °C) to remove any moisture. Then, the dried GNFs were transferred to a custom-built stirring sample holder. The Ru depositions were conducted at room temperature with a working pressure of 3 mTorr. Ar plasma was applied to a Ru target (Kurt J Lesker, 99.99%) with a current of 100 mA for 30 minutes, depositing from a distance of 110 mm. The resulting material was checked for homogeneity and sieved. Inductively coupled plasma-optical emission spectroscopy (ICP-OES) measurements were performed in triplicate on a PerkinElmer Optima 2000 spectrometer. The Ru/GNF catalyst (~10 mg) underwent microwave-assisted digestion using aqua regia (2mL) before being diluted to 10 mL with a 5% HCl solution. Loading was found to be 0.8 wt% Ru.

80 mg of GNF was uniformly spread onto a carbon sample holder, transferred into the main chamber, and evacuated to a base pressure of 1 × 10⁻⁷ Torr. Nitrogen gas was then introduced until a working pressure of 40 mTorr was reached. Nitrogen plasma treatment was performed by applying 100 W RF power to the sample holder for 60 s, with rotation at 30 rpm to ensure uniform ion exposure. This preparation was repeated in separate batches until a total of 200 mg of N-GNF was obtained. The N doping was confirmed by X-ray photoelectron spectroscopy (XPS, Figure S15). The resulting material was subsequently subjected to the same Ru deposition protocol describe for pure GNF.

**XPS:** X-ray photoelectron spectroscopy (XPS) was performed at one of the permanently mounted ultra-high vacuum endstations on the I09 beam line^[1]^ at Diamond Light Source. The I09 beamline consists of two light sources that cover soft (0.11 – 1.8 keV) and hard (2.1 – 15 keV) X-ray ranges. Each source has its own dedicated undulator and monochromator (soft: plane grating monochromator; hard: double crystal monochromator) and converge at the same point on the sample in the utilised endstation. Hard (HAXPES) X-ray photoelectron spectroscopy were acquired using a Scienta EW4000 HAXPES hemispherical energy analyser. The analyser was mounted perpendicular to the incident photons, in the plane of the photon polarisation (linear horizontal). The beam size of approximately 400 x 400 µm. HAXPES measurements were calibrated against the Au 4f peak (84.0 eV) from bulk Au foil. A photon energy of 2500 eV was used. XPS was also performed at the Nanoscale and Microscale Research Centre (nmRC) using a Thermo Fisher K-Alpha Photoelectron Spectrometer equipped with monochromatic Al Kα radiation source operating at 72 W (6 mA × 12 kV). The analysis area was approximately 400 × 600 microns, and all experimental conditions are detailed in Table S2. All samples were analysed using a dual ion-electron charge compensation detector, operating at an argon background pressure of 10⁻⁷ mbar. Samples were mounted by pressing them onto silicone-free, double-sided adhesive tape. Data processing was conducted using CASAXPS (Version 2.3.27), with charge correction applied to the reference C 1s peak.

**Table S2**. Experimental parameters used for the XPS measurements.

| Region | Pass Energy (eV) | Dwell time (s) | Number of scans | Step energy (eV) |
| --- | --- | --- | --- | --- |
| Survey | 160 | 150 | 2 | 0.2 |
| C 1s | 50 | 150 | 10 | 0.1 |
| N 1s | 50 | 150 | 50 | 0.1 |

**XAS:** Extended X-ray absorption fine structure (EXAFS) was performed on the Ru K-edge in transmission mode at the B18 beamline at the Diamond Light Source in Oxford, UK.^[2]^ Transmission spectra covering both the EXAFS region (200 eV below the edge to 850 eV above the edge) were collected in continuous scan mode with an acquisition time of approx. 90 seconds per spectrum. The X-ray energy was selected using a double crystal Si(111) monochromator, and spectra of a Ru metal foil were measured simultaneously for energy calibration. Data treatment was carried out in Athena from the Demeter software package.^[3]^

**AC-STEM:** AC-STEM imaging of nanocluster size and atomic structure were characterized by a JEOL JEM2100F aberration-corrected scanning transmission electron microscope (AC-STEM) equipped with a Cs probe corrector (CEOS) at a convergence angle of 19 mrad and annular dark field detector (ADF) operating with an inner angle of 31 mrad and outer angle of 82 mrad at 200 kV. The bright field (BF) detector was also used in parallel. Typically, samples were prepared dry, where samples were shaken in a vial containing the lacey carbon film Cu TEM grids (Agar Scientific). *In-situ* AC-STEM analysis of nanoclusters during NH_3_ decomposition was conducted on a JEOL ARM200CF optimised for atomic resolution spectroscopy. The microscope was operated in STEM mode at 80 kV utilizing a DENS *in-situ* gas cell system allowing for simultaneous heating, gas flow and mass spectrometry analysis. Samples were plasma cleaned in 5% O_2_ in Ar for 3 minutes and prepared dry without solvent dispersion. Images were first taken at 200 °C under 5 % H_2_ followed by heating to 450 °C under H_2_ for 1 h, then flowing 5 % NH_3_ for 1 h, taking images at each step.

**Computation Details:** We employed the Vienna Ab-initio Simulation Package (VASP) to model the effect of nitrogen coverage on its energy binding and N_2_ evolution on a periodic HCP Ru(0001) surface, simulating the terrace of the observed particles. The spin-polarised revised Perdew-Burke-Ernzerhof (RPBE) method of the generalised gradient approximation (GGA) was adopted to describe the exchange and correlation energies with a plane-wave kinetic cutoff of 500 eV.^[4]^ Spin alignments were fully relaxed to the lowest energy configuration, avoiding any constraint to the system.^[5]^ Non-spherical contributions to atomic cores from the electron density gradient were represented by the projector augmented wave (PAW).^[6]^ The zero-damping DFT-D3 method was used to describe long-range interactions.^[7]^ The optimised convergence thresholds of internal forces and electronic relaxation were set to 0.03 eV/Å and 10^−5^ eV, respectively. A 0.2 Å^−1^ k-spacing grid sampled the Brillouin zone with a smearing broadening of 0.2 eV using the Methfessel-Paxton method of first order.^[8]^

All surfaces were represented by a p(3 × 3) supercell slab model with five atomic layers, where the top three layers were fully relaxed and the bottom two were fixed at the optimised bulk lattice.^[9]^ We added 20 Å of vacuum perpendicular to the slab to avoid spurious interaction with periodic images. Dipole correction perpendicular to the surface was applied upon N adsorptions.

Nitrogen atoms were added sequentially to the surface, considering all the available sites. The most stable configuration was taken forward to explore the following adsorption site. A similar procedure was followed to investigate the nitrification of the Ru cluster. Nitrogen adsorption and cluster nitrification were considered energetically reversible for N desorption and denitrification, respectively.

The strength of atomic nitrogen binding ($E_{b}$) on the Ru(0001) surface was evaluated using Equation 3.

$$\begin{aligned} E_{b}=\frac{\left( E_{n\cdot N/Ru}-\left( E_{Ru}-n\cdot\frac{1}{2}E_{N_{2}} \right) \right)}{n} \#\left( Equation 3 \right) \end{aligned}$$

Where $E_{n\cdot N/Ru}$ is the energy of the structure containing $n$ nitrogen atoms, $E_{Ru}$ is the energy of the bare Ru surface, and $E_{N_{2}}$ is the energy of an isolated nitrogen molecule, calculated using a broken symmetry cubic simulation cell with a 15 Å lattice. The N_2_ evolution reaction was characterised by an energy profile, whose relative energy ($\Delta E$) was calculated according to Equation 4, where $m$ indicates the number of N_2_ molecules desorbed and $n_{T}$ is the total number of nitrogens on the structure (8 in the case represented here). Equation 5 gives the reaction energy ($E_{r}$) of each step as the energy difference between the final state (FS), e.g. 6·N/Ru(0001), and the initial state (IS), e.g. 8·N/Ru(0001). An exothermic reaction step is associated with a negative $E_{r}$. The transition states (TS) were determined using the climb-image nudged elastic band (ci-NEB) combined with the improved dimer method (IDM), ensuring a unique imaginary frequency along the reaction coordinate.^[10–12]^ Vibrational frequency calculations were carried out by constructing and diagonalising the Hessian matrix, built from finite displacements of atomic positions of 0.05 Å in length. Only displacements of the adsorbates were regarded, i.e. the adsorbate vibrations were treated decoupled from the surface, since test calculations considering them yielded negligible changes in the gained vibrational frequencies at an exceedingly high computational cost. We defined the activation energy ($E_{a}$) as the energy difference between TS and IS energies (Equation 6).

$$\begin{aligned} \Delta E=\left( m\cdot{E_{N}}_{2}+E_{n\cdot N/Ru} \right)-\left( n_{T}\cdot\frac{1}{2}{E_{N}}_{2}+E_{Ru} \right)\#\left( Equation 4 \right) \end{aligned}$$

$$\begin{aligned} E_{r}=E^{\mathrm{FS}}-E^{\mathrm{IS}} \#\left( Equation 5 \right) \end{aligned}$$

$$\begin{aligned} E_{a}=E^{\mathrm{TS}}-E^{\mathrm{IS}}\#\left( Equation 6 \right) \end{aligned}$$

**Table S3**. Energy values of each step of the atomic nitrogen recombination along the energy profile in Figure 2b. All values are relative to the same energy reference, i.e. the energy of four N_2_ molecules and a bare Ru(0001) slab.

| **System** | **ΔE (eV)** |
| --- | --- |
| $\boldsymbol{8}\boldsymbol{N}^{\boldsymbol{*}}\boldsymbol{/Ru(0001)}$ | 1.367 |
| Transition State | 2.876 |
| $\boldsymbol{(N}_{\boldsymbol{2}}^{\boldsymbol{*}}\boldsymbol{+6}\boldsymbol{N}^{\boldsymbol{*}}\boldsymbol{)}\boldsymbol{/Ru(0001)}$ | -1.013 |
| $\boldsymbol{N}_{\boldsymbol{2}}\boldsymbol{(g)+6}\boldsymbol{N}^{\boldsymbol{*}}\boldsymbol{/Ru(0001)}$ | -0.896 |
| Transition State | 0.711 |
| $N_{2}\left( g \right)\boldsymbol{+}\boldsymbol{(N}_{\boldsymbol{2}}^{\boldsymbol{*}}\boldsymbol{+}4\boldsymbol{N}^{\boldsymbol{*}}\boldsymbol{)}\boldsymbol{/Ru(0001)}$ | -2.016 |
| $2N_{2}(g)\boldsymbol{+}4N^{*}\boldsymbol{/Ru(0001)}$ | -1.949 |
| Transition State | 0.156 |
| ${2N_{2}\left( g \right)+\boldsymbol{(N}}_{\boldsymbol{2}}^{\boldsymbol{*}}\boldsymbol{+}2\boldsymbol{N}^{\boldsymbol{*}}\boldsymbol{)}\boldsymbol{/Ru(0001)}$ | -2.115 |
| $3N_{2}(g)\boldsymbol{+}2\boldsymbol{N}^{\boldsymbol{*}}\boldsymbol{/Ru(0001)}$ | -1.563 |
| Transition State | 1.077 |
| $3N_{2}(g)\boldsymbol{+}\boldsymbol{N}_{2}^{\boldsymbol{*}}\boldsymbol{/Ru(0001)}$ | 0.263 |
| $4N_{2}\left( g \right)\boldsymbol{+Ru(0001)}$ | 0.000 |

**i)**

**Figure S1** Arrhenius plots of Ru/GNF after 3, 8, 30 & 100 h reaction times.

**Figure S2** High magnification AC-STEM images of Ru/GNF nanoparticles **a-c)** after reduction **d-f)** after 3 h NH_3_ decomposition and **g-i)** after 60 h NH_3_ decomposition.


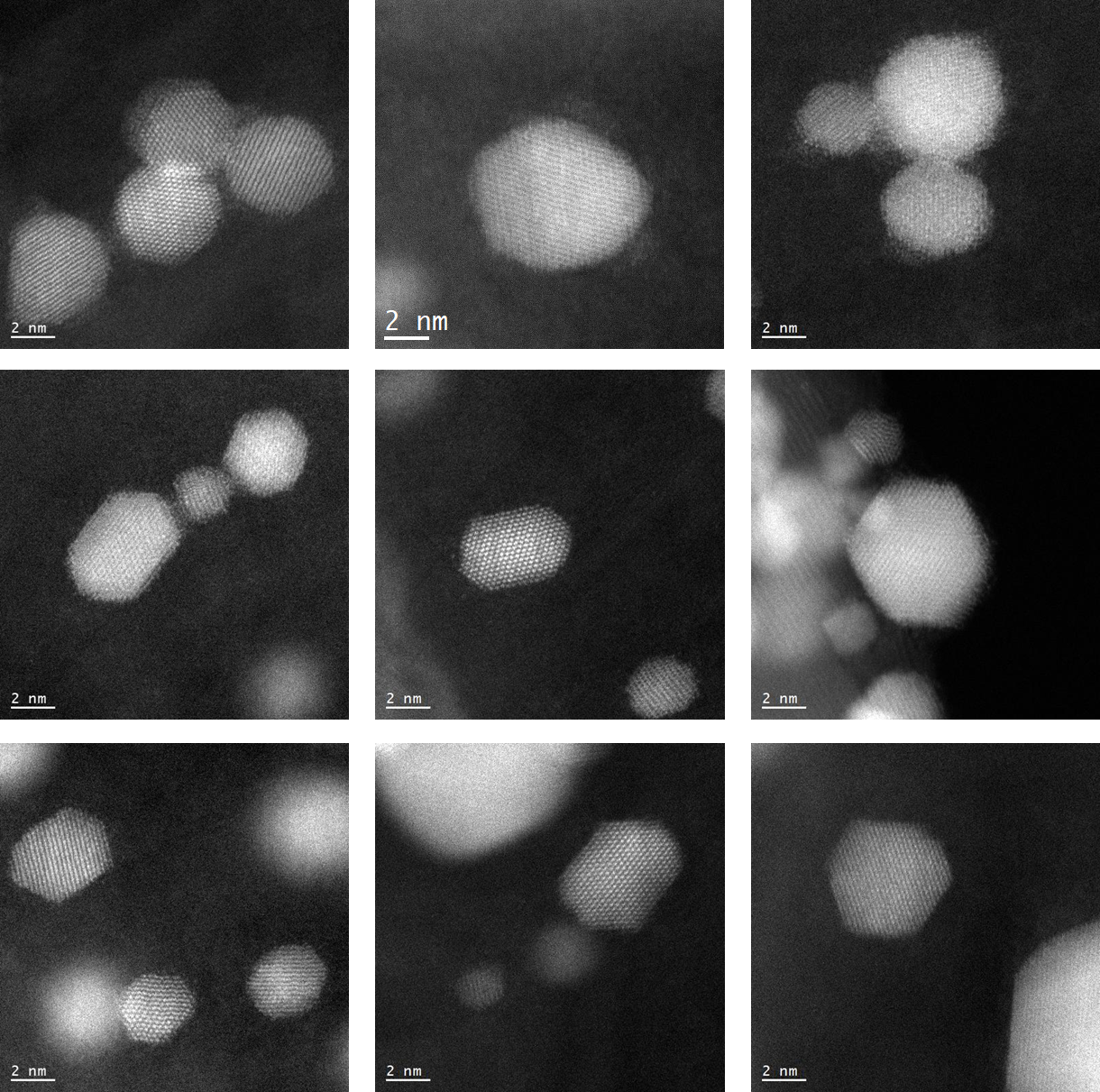


**a)**

**c)**

**d)**

**e)**

**f)**

**g)**

**h)**

**b)**


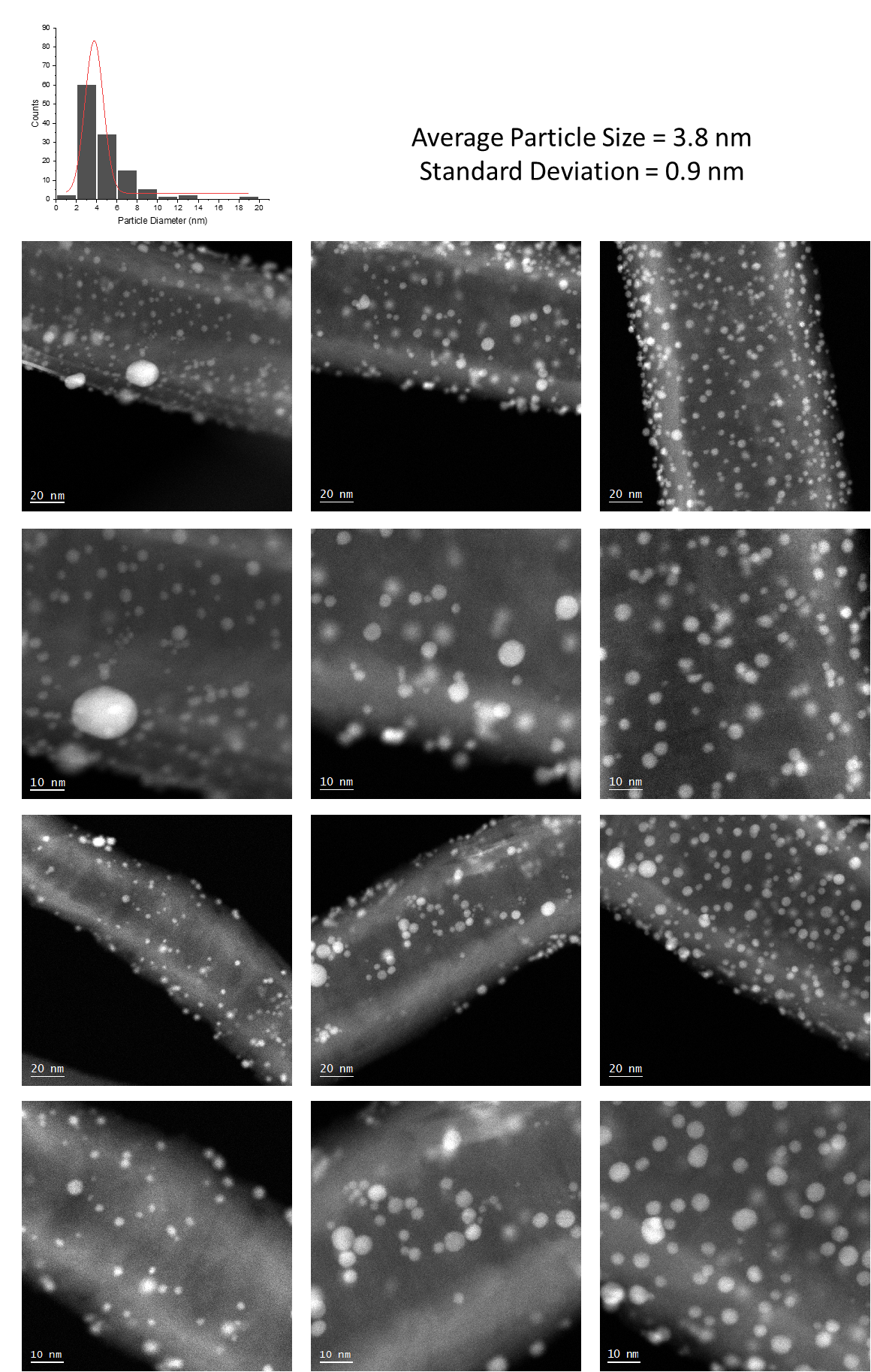


**Figure S3** AC-STEM images used for particle size analysis of Ru/GNF after reduction conditions. Rows a) and c) are 1 Mx magnification and rows b) and d) are their respective 2 Mx magnification.

**a)**

**b)**

**c)**

**d)**


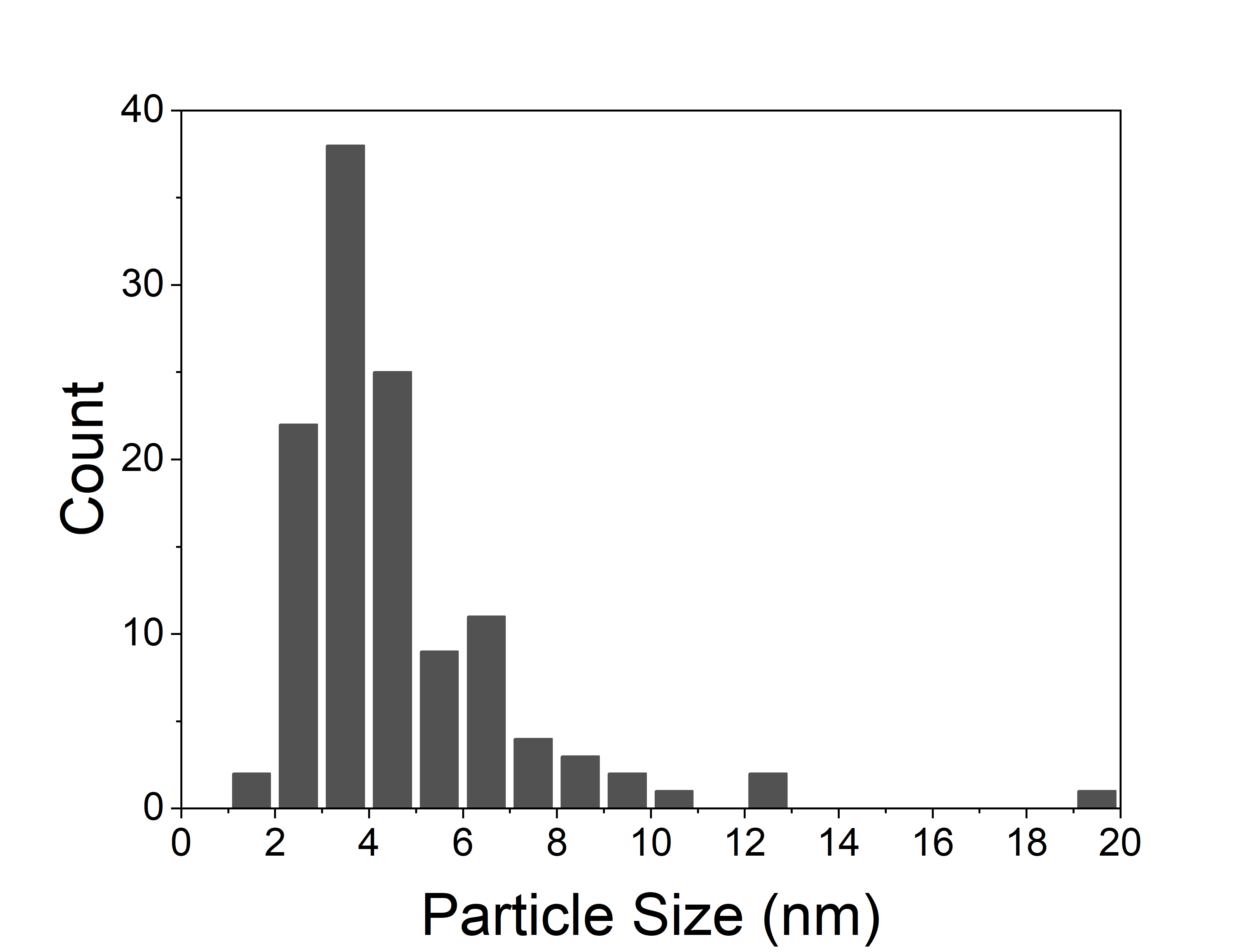

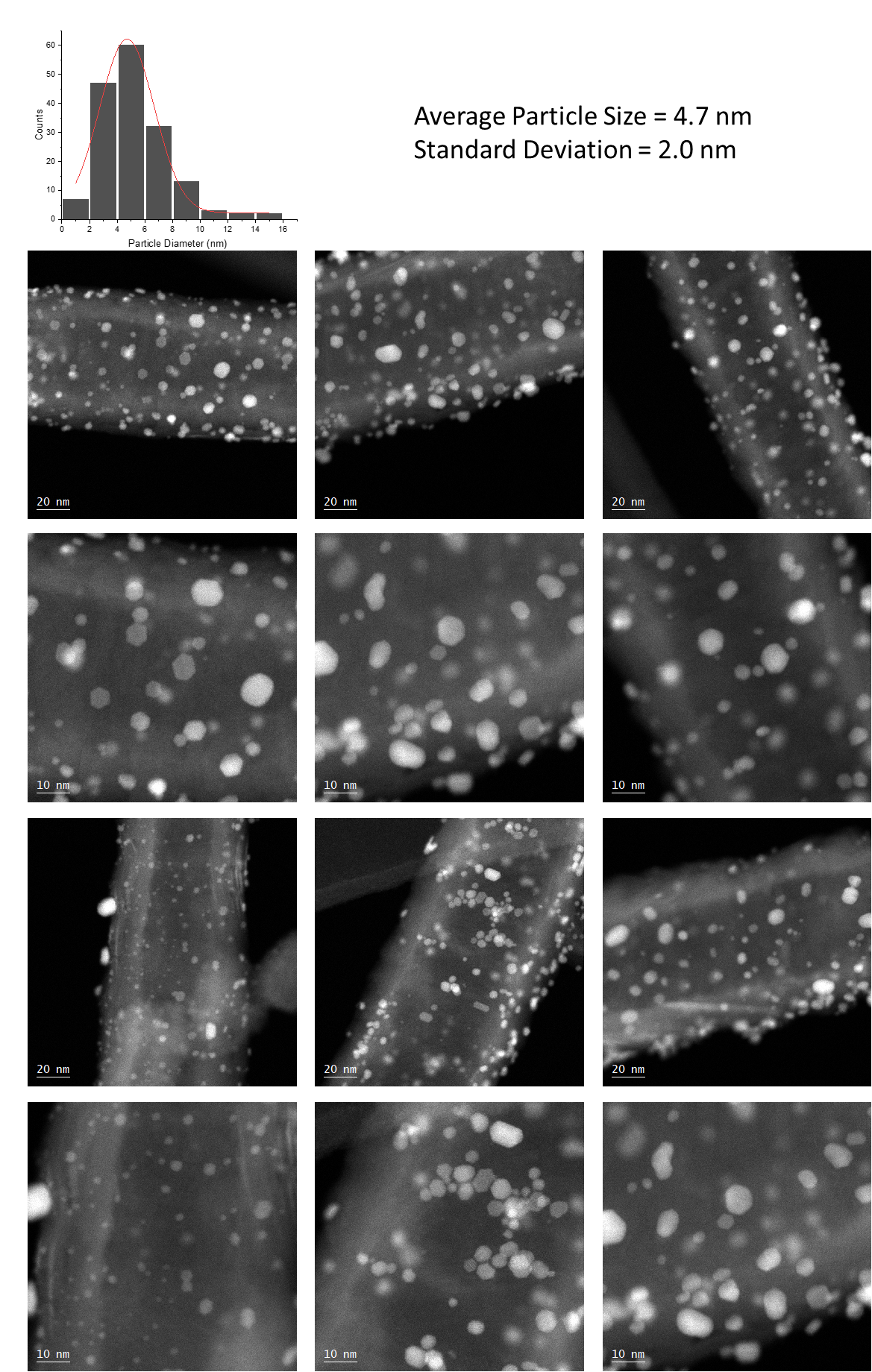


**Figure S4** AC-STEM images used for particle size analysis of Ru/GNF after 3 h reaction. Rows a) and c) are 1 Mx magnification and rows b) and d) are their respective 2 Mx magnification.

**b)**

**a)**

**c)**

**d)**


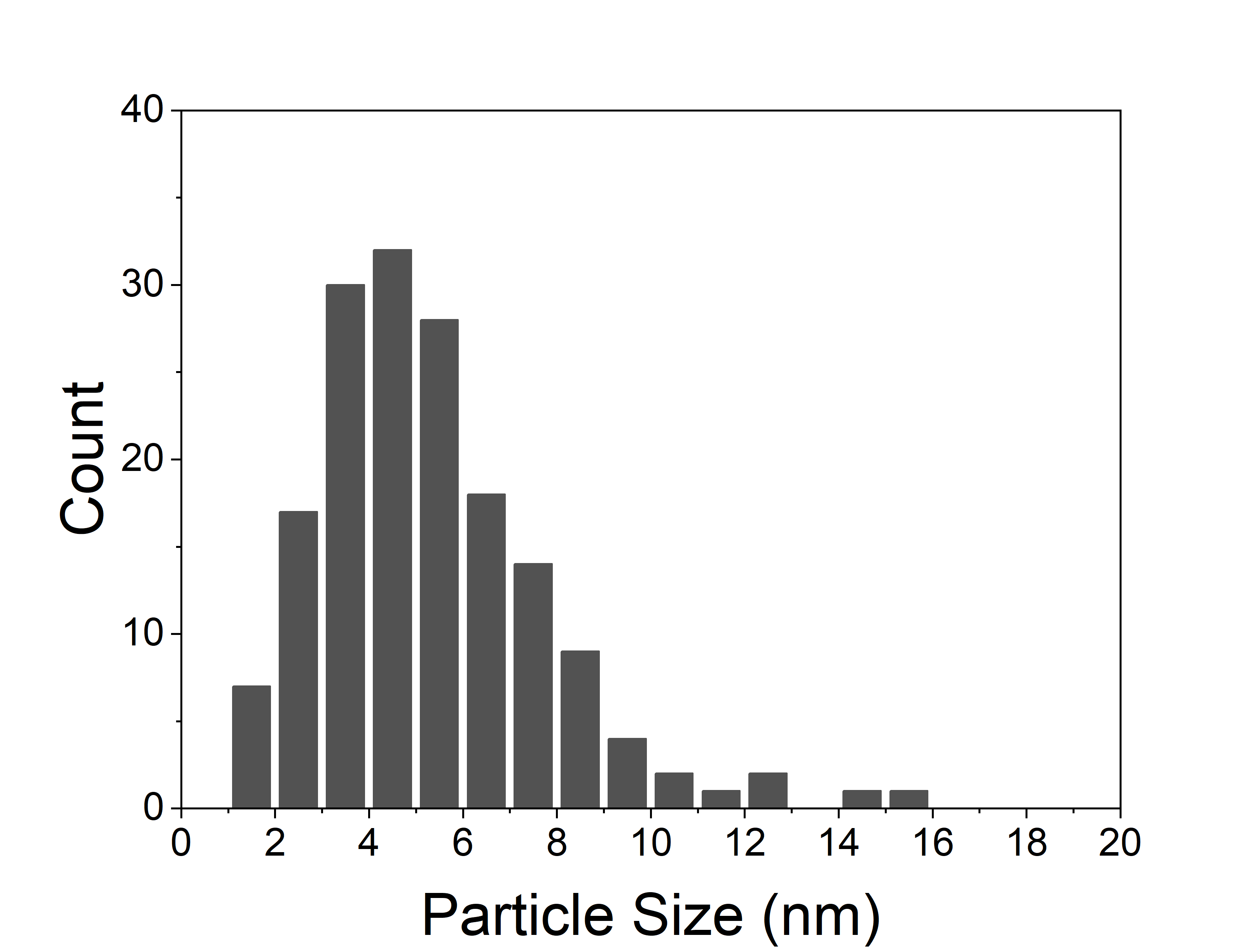

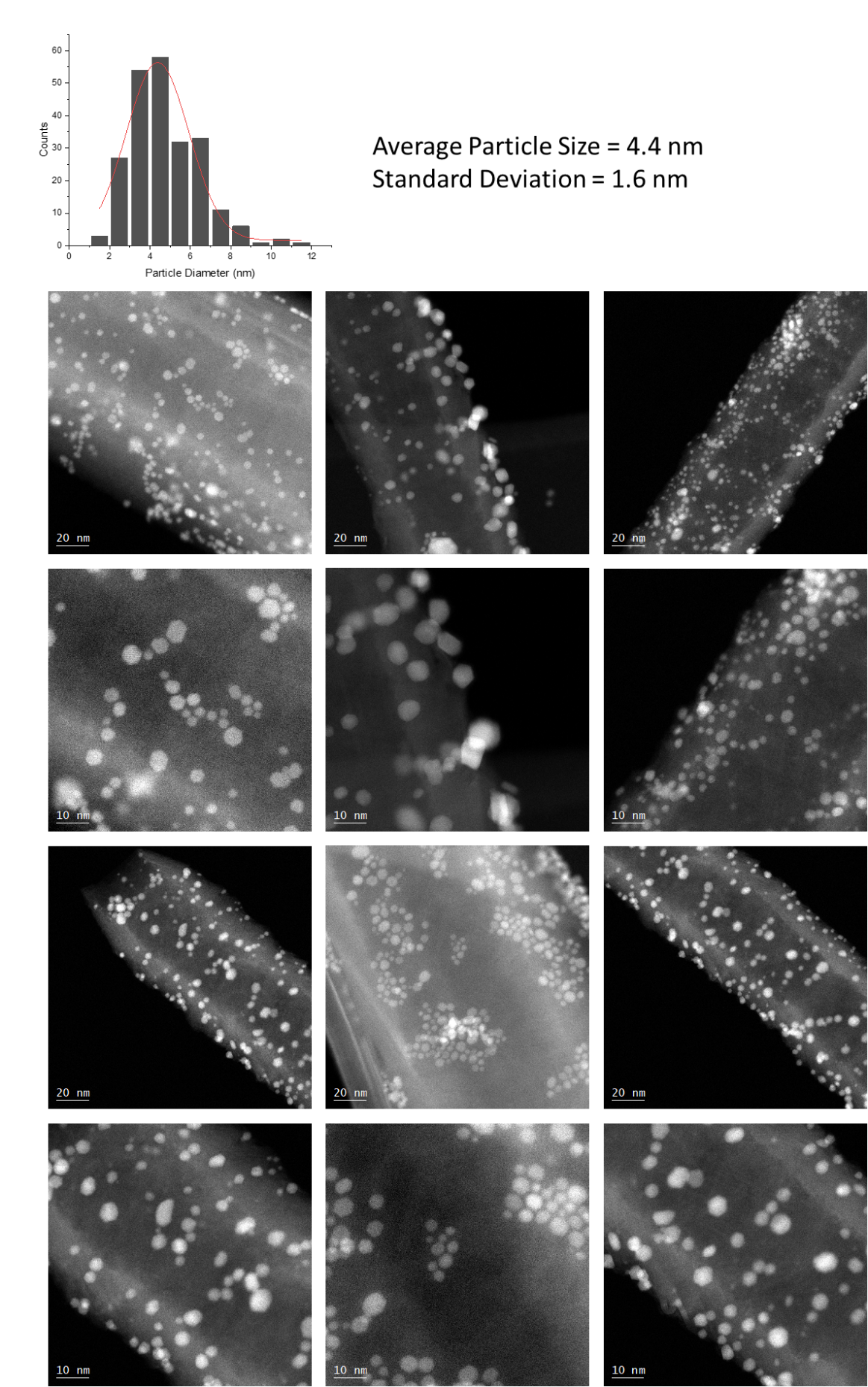


**Figure S5** AC-STEM images used for particle size analysis of Ru/GNF after 60 h reaction. Rows a) and c) are 1 Mx magnification and rows b) and d) are their respective 2 Mx magnification.

**a)**

**b)**

**c)**

**d)**


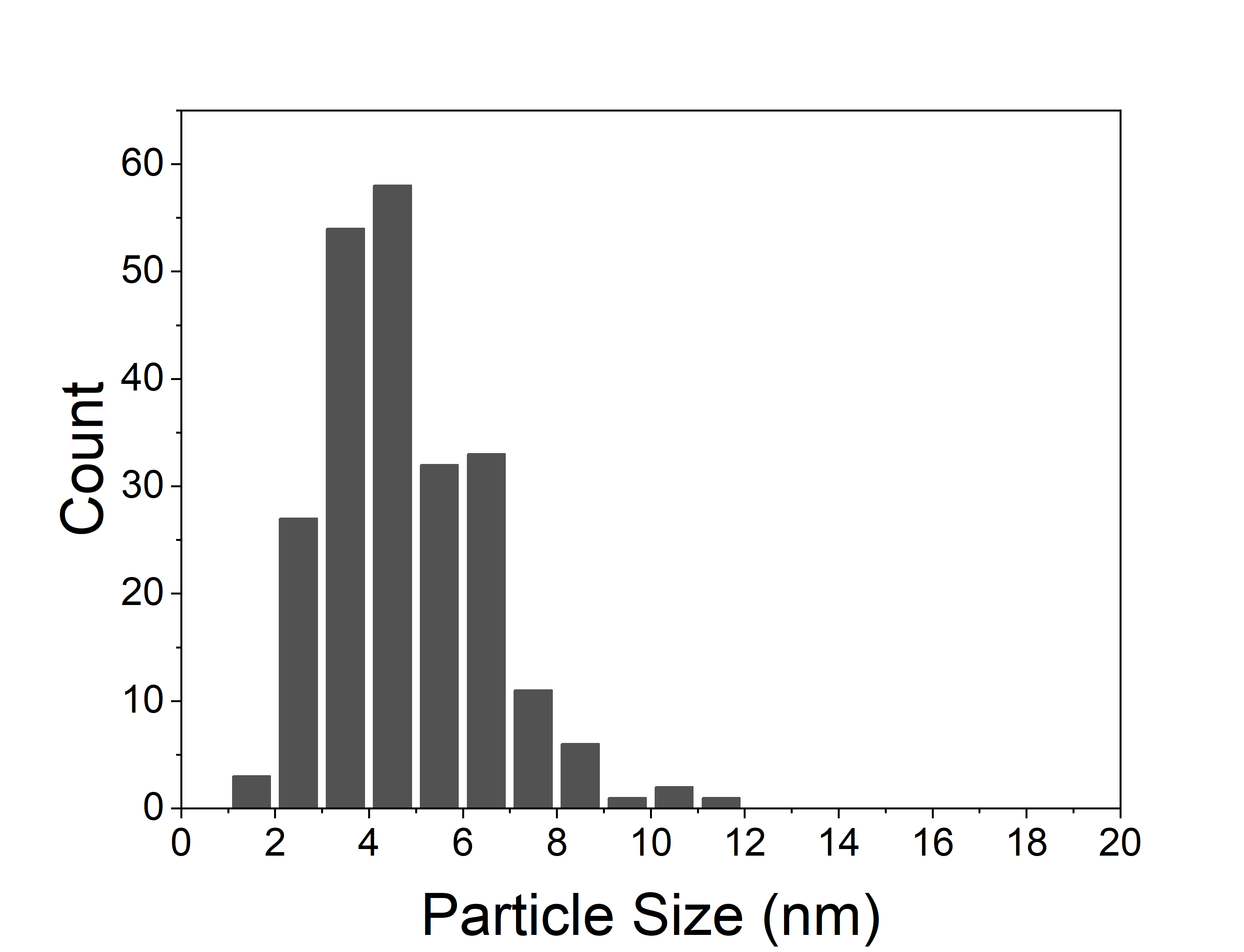

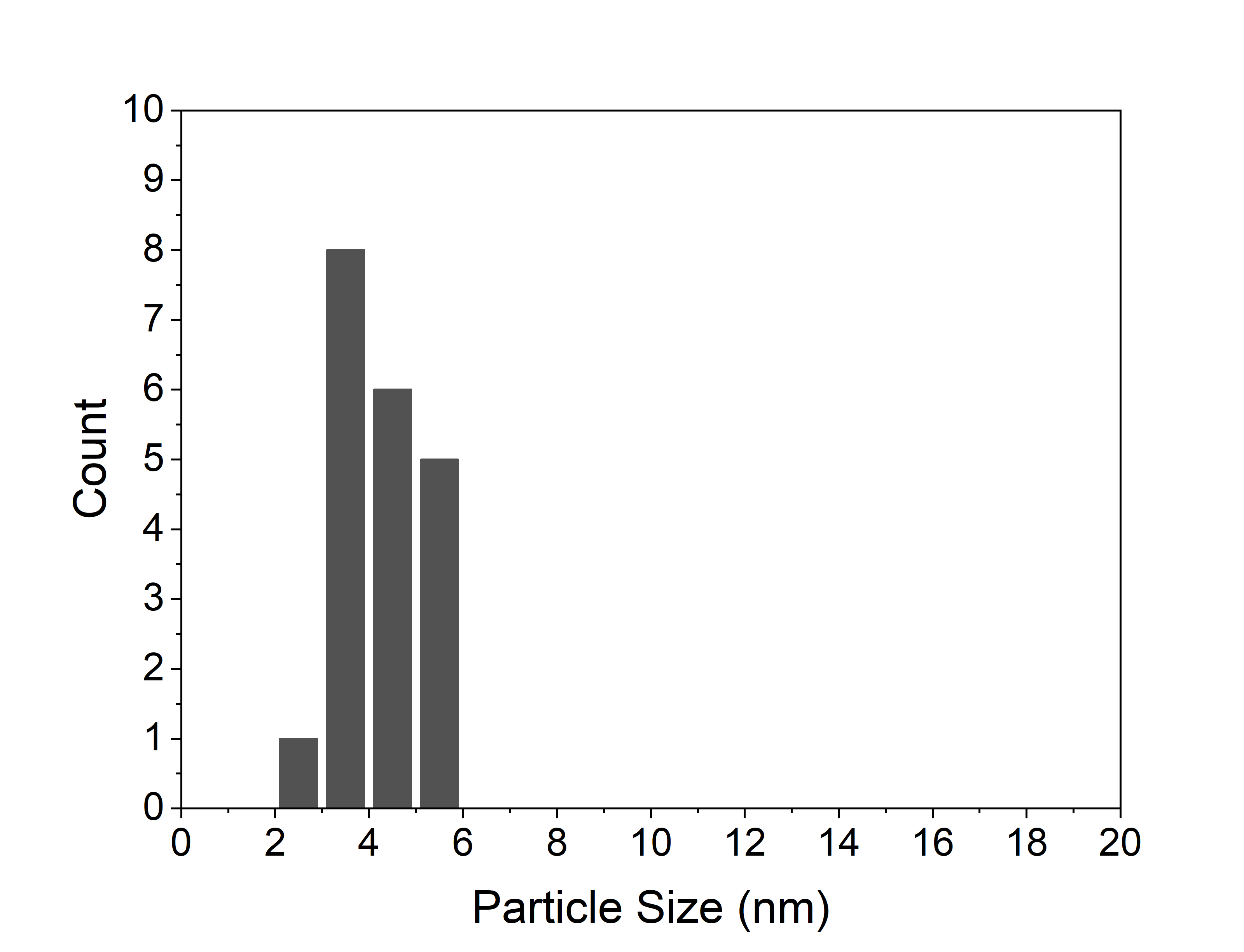

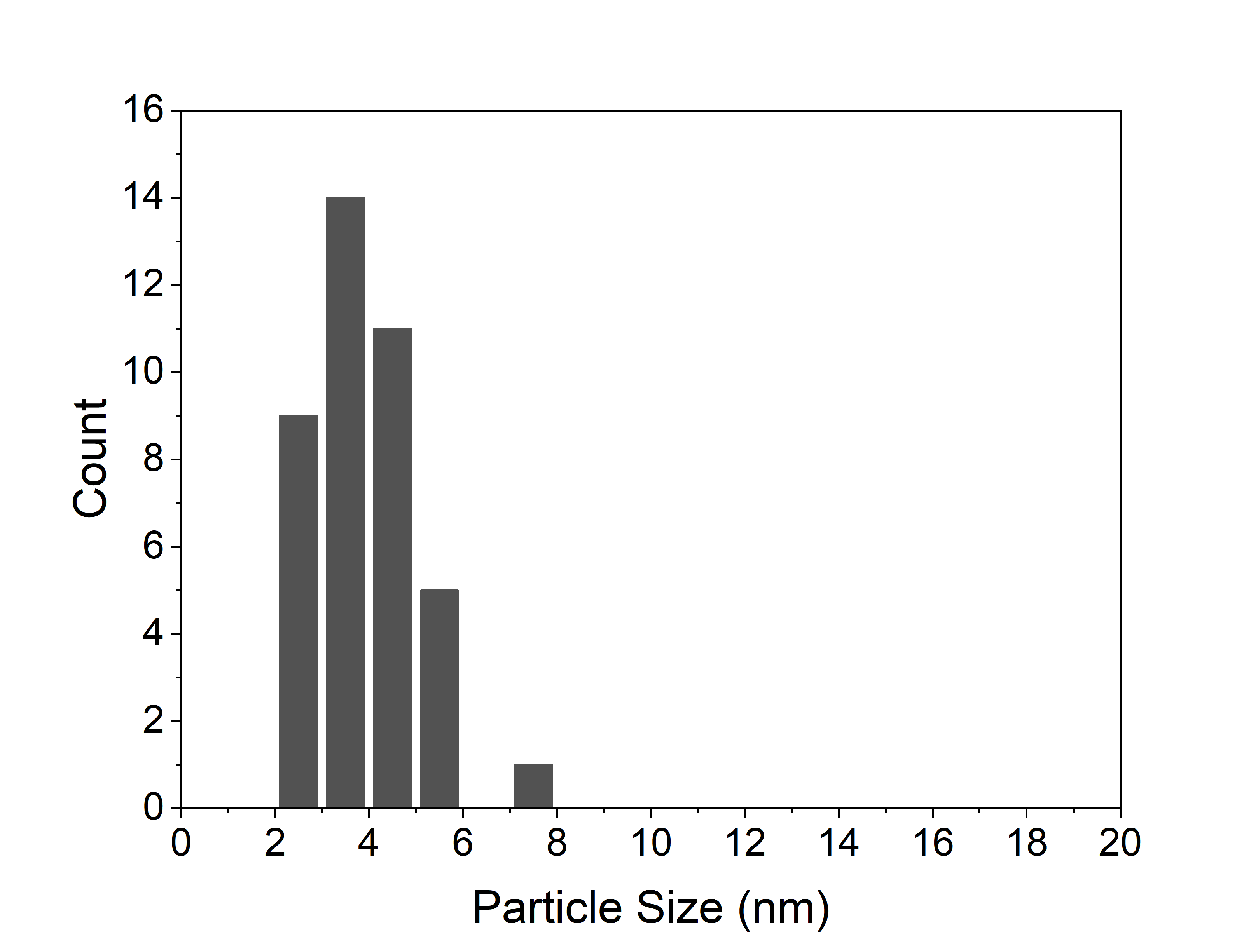


**Figure S6** In-operando AC-STEM images of a) <200 °C under 5 % H_2_ b) 450 °C 5% H_2_ after 1 h with its respective particle distribution below c) 450 °C 5 % NH_3_ after 1 h with its respective particle distribution below and d) Image of the deposition of Ru/GNF on to the DENS in-situ gas cell chip before insertion in to microscope, the grooves allow for controlled gas flow across the sample.


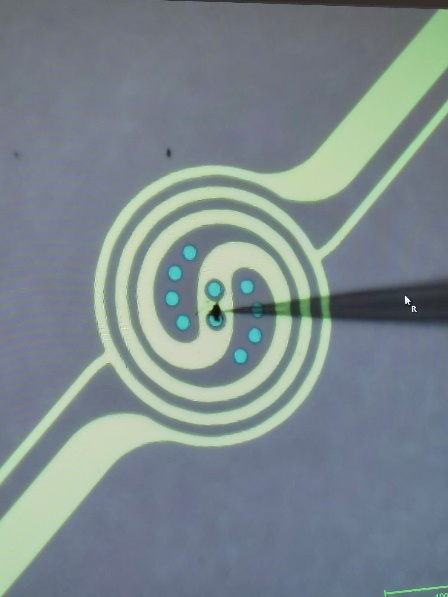


a)

b)

c)

d)

Average Particle Diameter = 3.7 nm

Standard Deviation = 1.2 nm

Average Particle Diameter = 3.9 nm

Standard Deviation = 0.6 nm


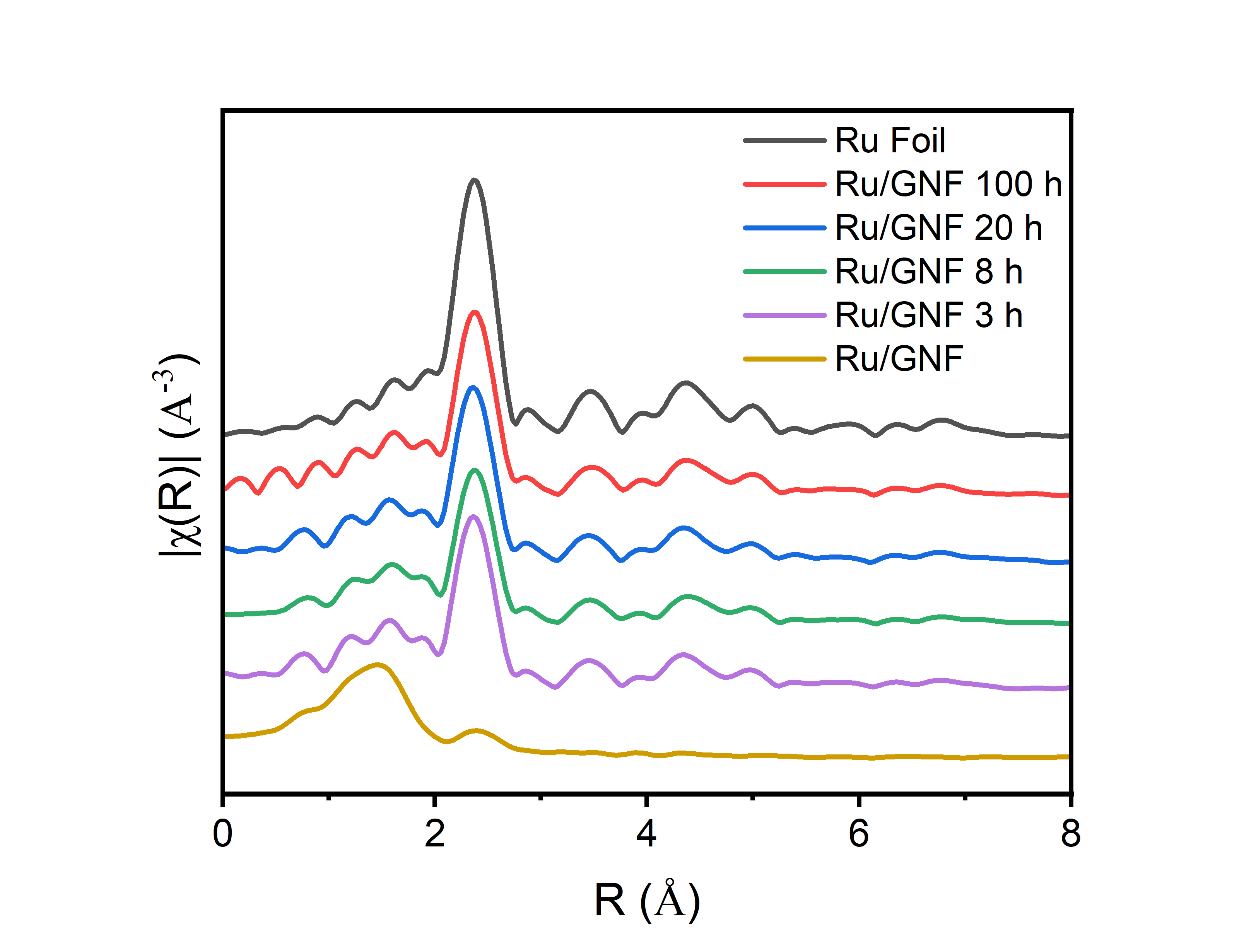

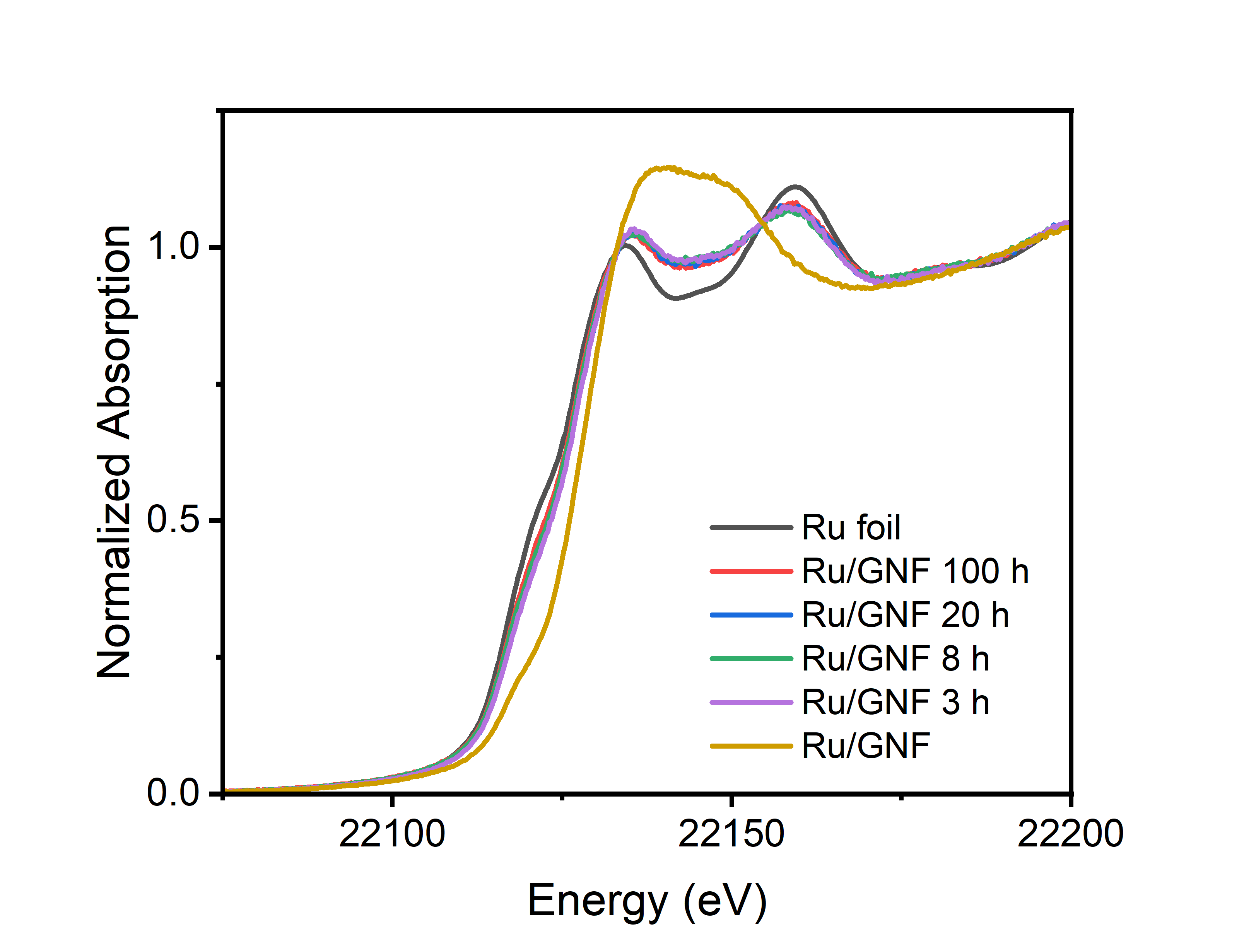


**Figure S7 a)** XANES analysis of fresh Ru/GNF (yellow), after 3h reaction (purple), after 8 h reaction (green), after 20 h reaction (blue), after 100 h reaction (red) and Ru foil. **b)** EXAFS analysis of fresh Ru/GNF (yellow), after 3h reaction (purple), after 8 h reaction (green), after 20 h reaction (blue), after 100 h reaction (red) and Ru foil.

**a)**

**b)**

**Figure S9** Hydrogen production from ammonia decomposition reaction of Ru/La_2_O_3_ (0.8 wt%) after a) 3 h of reaction b) 10 h of reaction. The corresponding Arrhenius plot and apparent activation energy was taken after c) 3h (E_a_ = 72.0 ± 5.6 kJ mol^-1^) and d) 10 h (E_a_ = 78.1 ± 4.6 kJ mol^-1^) of reaction.











**a)**

**b)**

**c)**

**d)**


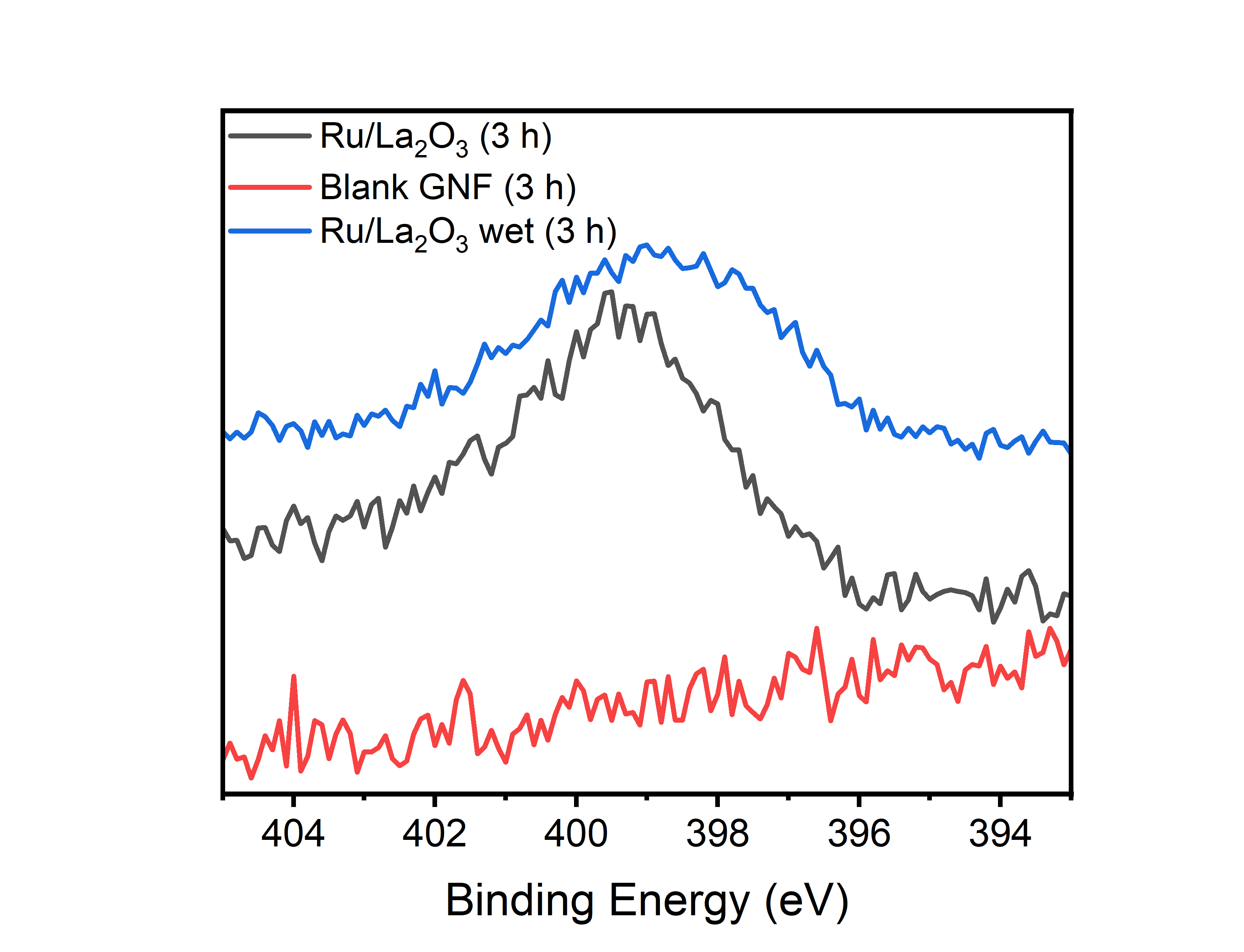

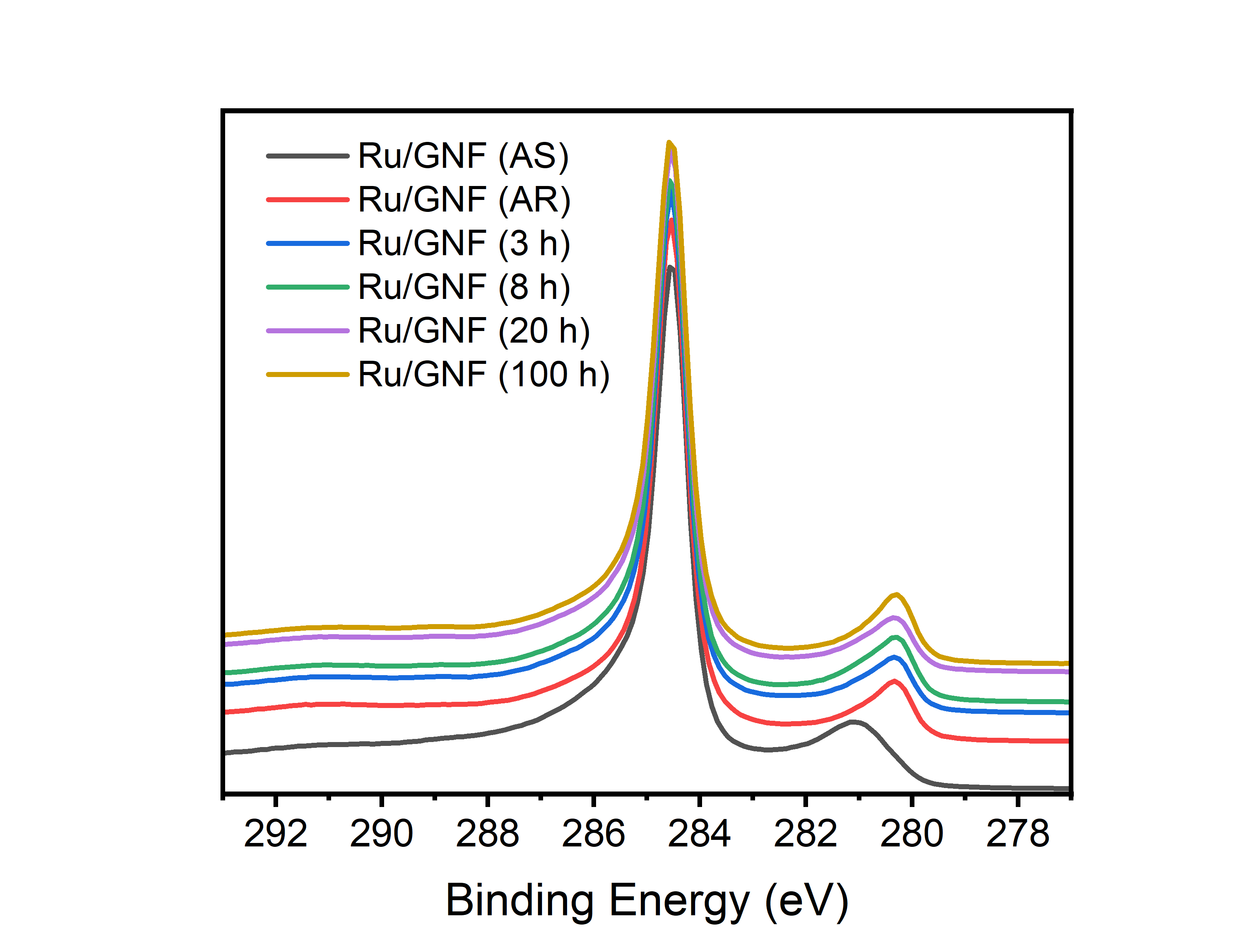


**Figure S8** XPS spectra of a) Ru 3d region of Ru/GNF as sputtered (AS), after reduction (AR) and after different reaction times. b) N 1s region of sputtered and wet impregnation prepared Ru/La_2_O_3_ & Blank GNF after 3 h reaction.

**b)**

**a)**


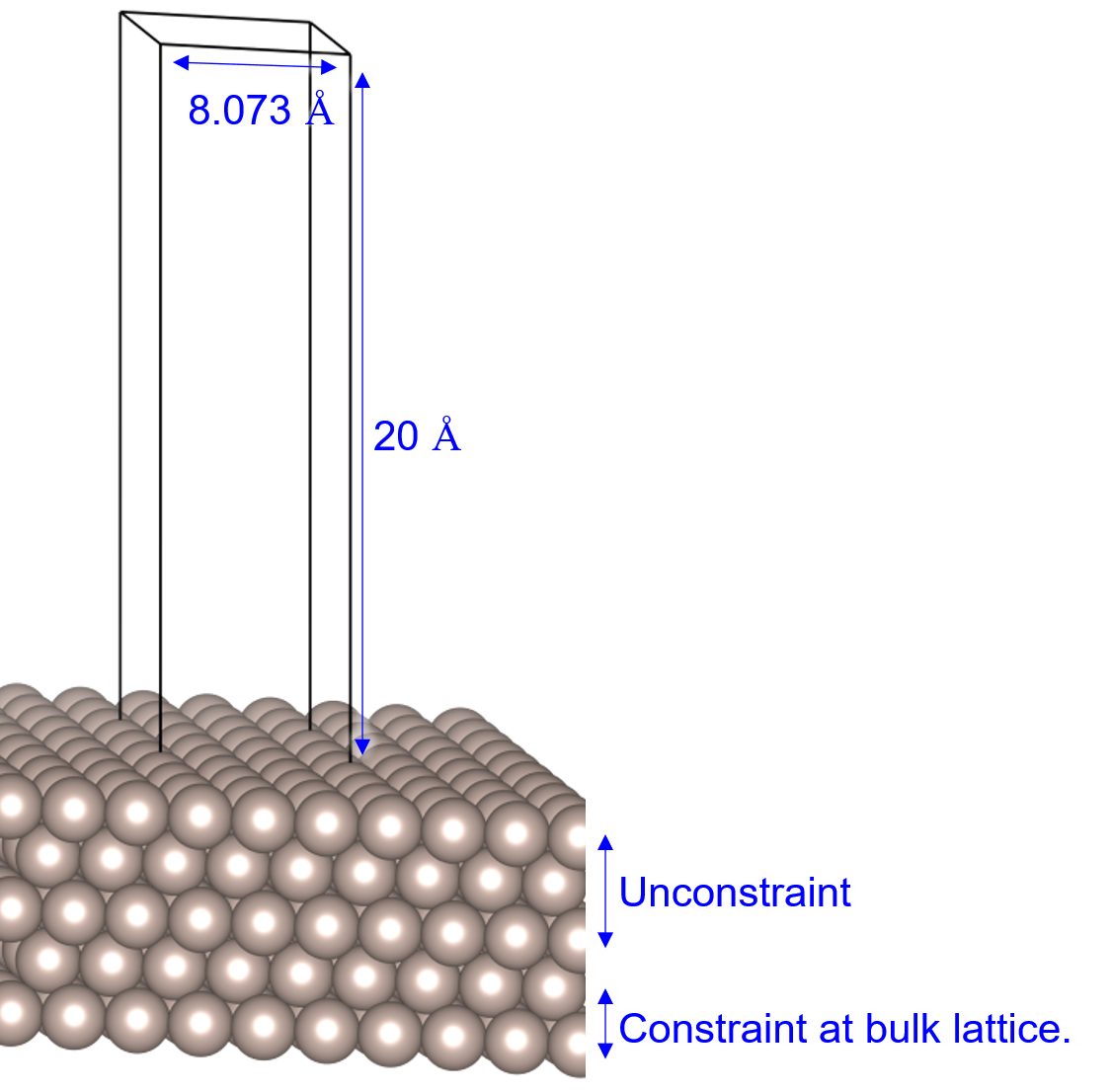


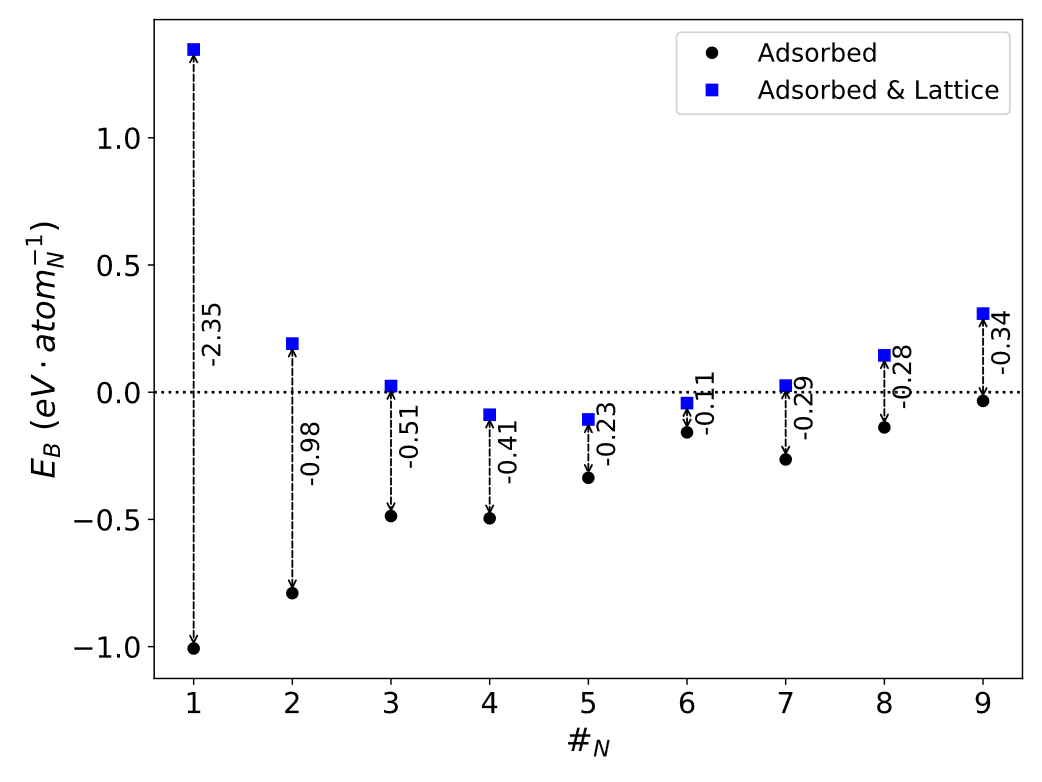


**Figure S10** The lowest binding energies (E_B_) of atomic nitrogen on the Ru(0001) slab (black circles) and with one of the N placed below the top Ru atomic layer (blue squares). Inset arrows and numbers indicate the energy required to stabilise an adsorbed N into the Ru lattice.

**Figure S11** Representation of the Ru(0001) periodic slab; the black frame indicates the simulation cell. Colour scheme: Grey indicates Ru atoms.


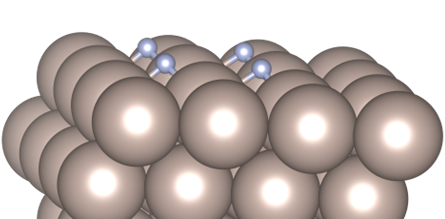

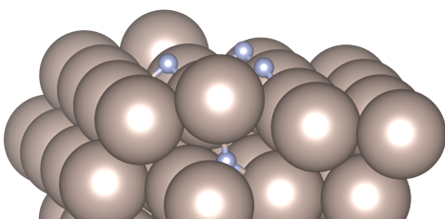


**Figure S12** Representation of exemplary 4 N atoms on Ru(0001) structures. The left image contains all atoms on the surface, while the right image contains a nitrogen atom in the Ru lattice. Colour scheme: Grey is Ru and pale blue is N.

**Figure S13** Extended temperature H_2_-TPSR data for Ru/GNF after 24 h reaction (blue) and 48 h reaction (black).


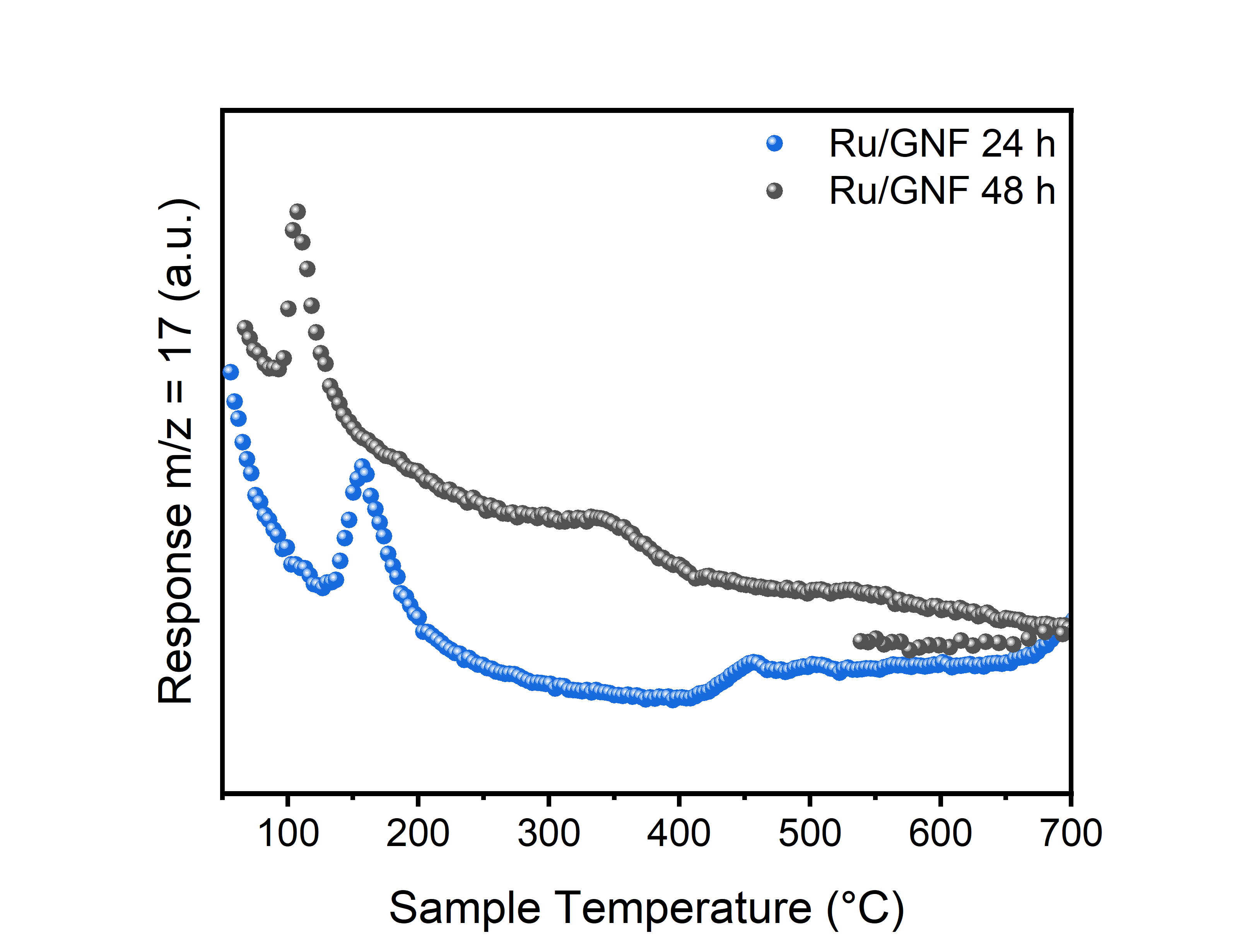


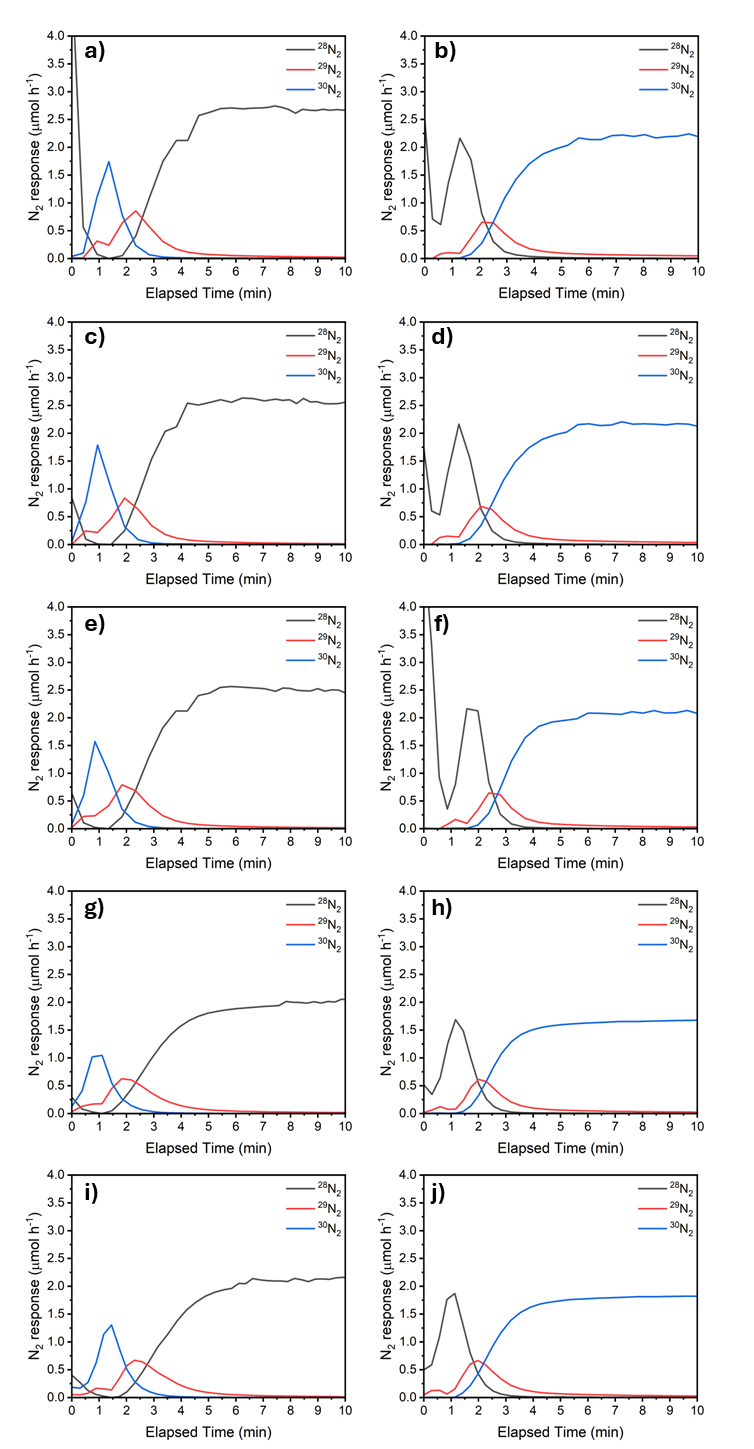


**Figure S14** Isotopic labelling results after flushing 5,7,10,13 and 16 minutes with He. Left panel (a,c,e,g and i) shows ^15^NH_3_ flow, followed by a He flush then introduction of ^14^NH_3_. Right panel (b,d,f,h and j) shows ^14^NH_3_ flow followed by a He flush then introduction of ^15^NH_3_.


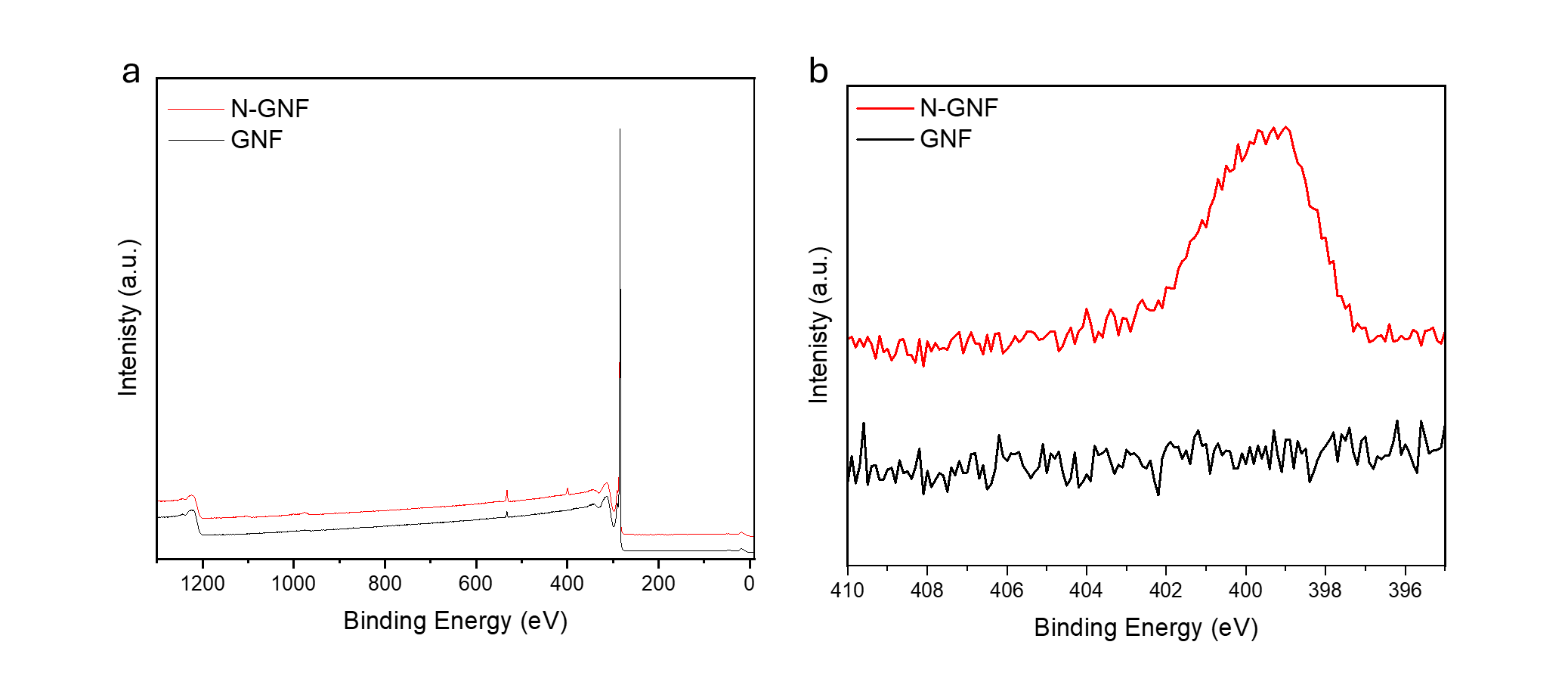


**Figure S15** Survey (a) and high-resolution N 1s (b) spectra for the N-GNF and pristine GNF samples. The N-GNF presenting 2% N/C ratio on the GNF lattice surface.


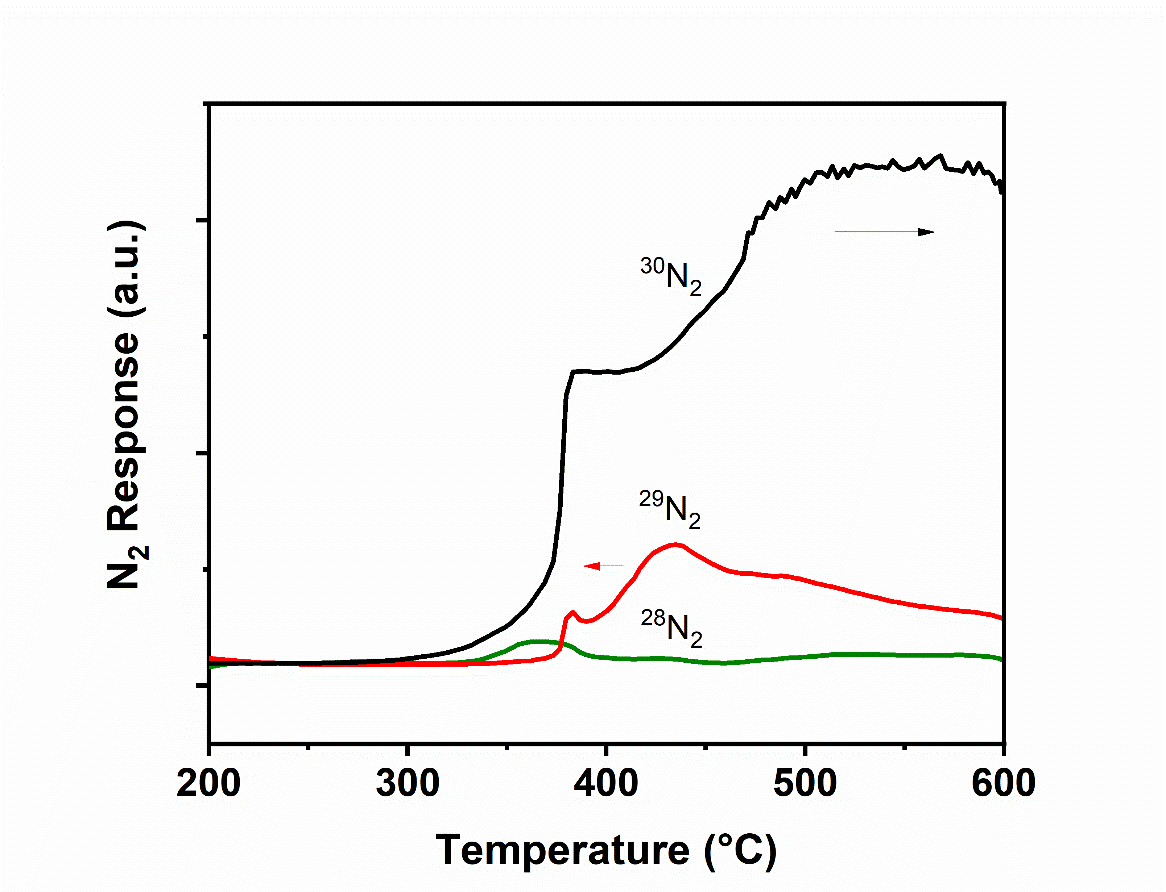




**a)**

**b)**

**Figure S16** a)^15^NH_3_ scrambling experiment with Ru on N-doped GNF (Ru/N-GNF). Where ^30^N_2_ (black), ^29^N_2_ (red) and ^28^N_2_ (green) are measured. b) Scrambling of pure N-GNF with ^15^NH_3_ after flushing with He, also showing a baseline response of ^28^N_2_ (black) and ^39^N_2_ (blue).

**Figure S17.** Isotopic Scrambling of ^28^N_2_ after 12 h of reaction with ^15^NH_3_ at 450 °C. Both ^29^N_2_ (black) and ^30^N_2_ (red) exhibit baseline response.


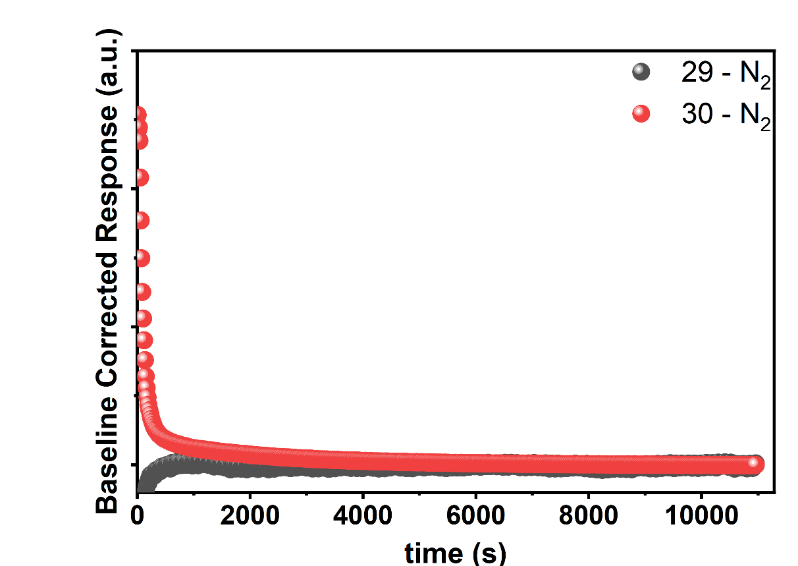

**Figure S18.** NH_3_ rate order for Ru/GNF after 40h at 450°C. A rate order of 0.7 is found.

**References**

[1] T.-L. Lee, D. A. and Duncan, “A Two-Color Beamline for Electron Spectroscopies at Diamond Light Source” *Synchrotron Radiation News* **2018**, *31*, 16–22.

[2] A. J. Dent, G. Cibin, S. Ramos, A. D. Smith, S. M. Scott, L. Varandas, M. R. Pearson, N. A. Krumpa, C. P. Jones, P. E. Robbins, “B18: A core XAS spectroscopy beamline for Diamond” *J. Phys.: Conf. Ser.* **2009**, *190*, 012039.

[3] B. Ravel, M. Newville, “ATHENA, ARTEMIS, HEPHAESTUS: data analysis for X-ray absorption spectroscopy using IFEFFIT” *J Synchrotron Rad* **2005**, *12*, 537–541.

[4] B. Hammer, L. B. Hansen, J. K. Nørskov, “Improved adsorption energetics within density-functional theory using revised Perdew-Burke-Ernzerhof functionals” *Phys. Rev. B* **1999**, *59*, 7413–7421.

[5] Q. Dang, S. Tang, T. Liu, X. Li, X. Wang, W. Zhong, Y. Luo, J. Jiang, “Regulating Electronic Spin Moments of Single-Atom Catalyst Sites via Single-Atom Promoter Tuning on S-Vacancy MoS2for Efficient Nitrogen Fixation” *Journal of Physical Chemistry Letters* **2021**, *12*, 8355–8362.

[6] D. Joubert, “From ultrasoft pseudopotentials to the projector augmented-wave method” *Physical Review B - Condensed Matter and Materials Physics* **1999**, *59*, 1758–1775.

[7] S. Grimme, S. Ehrlich, L. Goerigk, “Effect of the damping function in dispersion corrected density functional theory” *Journal of Computational Chemistry* **2011**, *32*, 1456–1465.

[8] M. Methfessel, A. T. Paxton, “High-precision sampling for Brillouin-zone integration in metals” *Physical Review B* **1989**, *40*, 3616–3621.

[9] X. Lu, J. Zhang, W. K. Chen, A. Roldan, “Kinetic and mechanistic analysis of NH3decomposition on Ru(0001), Ru(111) and Ir(111) surfaces” *Nanoscale Advances* **2021**, *3*, 1624–1632.

[10] G. Henkelman, B. P. Uberuaga, H. Jonsson, H. Jónsson, “A climbing image nudged elastic band method for finding saddle points and minimum energy paths” *The Journal of Chemical Physics* **2000**, *113*, 9901–9904.

[11] G. Henkelman, H. Jónsson, “A dimer method for finding saddle points on high dimensional potential surfaces using only first derivatives” *The Journal of Chemical Physics* **1999**, *111*, 7010–7022.

[12] G. Henkelman, H. Jonsson, H. Jónsson, “Improved tangent estimate in the nudged elastic band method for finding minimum energy paths and saddle points” *The Journal of Chemical Physics* **2000**, *113*, 9978–9985.
